# Assessing the treatment of pancreatic ductal adenocarcinoma by deuterium metabolic imaging: a preclinical study

**DOI:** 10.1007/s10334-026-01340-z

**Published:** 2026-04-06

**Authors:** Elton T. Montrazi, Maya Kovalevsky, Keren Sasson, Lilach Agemy, Avigdor Scherz, Lucio Frydman

**Affiliations:** 1 Department of Chemical and Biological Physics, Weizmann Institute of Science, Rehovot, Israel; 2 Department of Plant and Environmental Sciences, Weizmann Institute of Science, Rehovot, Israel

**Keywords:** Pancreatic cancer, 2H MRSI, Anaerobic glycolysis, Warburg effect, Cyclophosphamide treatment, Biomarking

## Abstract

**Purpose:**

To evaluate the utility of Deuterium Metabolic Imaging (DMI) as a tool for monitoring disease progression and assessing the therapeutic efficacy of cyclophosphamide in pancreatic ductal adenocarcinoma (PDAC).

**Methods:**

The study utilized C57BL mice implanted with a PDAC model, and separated into two experimental groups: One cohort served as an untreated control, while the second was treated with the chemotherapeutic agent cyclophosphamide (CP). Metabolic mapping for the two cohorts was performed over the course of weeks using 2H MRSI at 15.2T, tracking the metabolic pathway of deuterated glucose as it converts by the PDAC into lactate.

**Results:**

CP administration led to a significant reduction in tumor growth and improved survival rates compared to the control group. Tumor growth rates for the untreated, control group, showed a strong inverse correlation for glucose’s uptake and a strong direct correlation with the glucose-to-lactate conversion rates as seen by DMI. CP treatment disrupted both these correlations: Post-treatment, tumor growth rates became statistically uncorrelated with either glucose consumption or lactate production which, when normalized for tumor size, remained relatively constant during the treatment period.

**Conclusion:**

The study demonstrates that CP treatment fundamentally alters the metabolic kinetics of glucose in PDAC tumors, inducing disruptions in metabolic correlations that may serve for distinguishing therapeutic success from failure.

**Supplementary Information:**

The online version contains supplementary material available at 10.1007/s10334-026-01340-z.

## Introduction

Pancreatic ductal adenocarcinoma (PDAC) is a leading cause of cancer-related mortality. [1] In Israel, the United States and in many European countries, pancreatic cancer is currently the 3rd most common cause of cancer-related deaths; [2] given present trends and the progress happening in the diagnosis of more widespread kinds of cancers—breast, prostate, lung, colon—PDAC is expected to become the second deadliest cancer during the 2030s. [3] PDAC’s five-year survival expectancy has improved over the last decades, but still stands at a dismal rate of ≈20%. One of the reasons making PDAC such a deadly cancer is the dearth of reliable early diagnosis methods. This is compounded by the paucity of treatment options, and of reliable reporters attesting, early into the treatment, the success or lack thereof for a chosen route of action. Thus, while diagnostic and treatment alternatives have improved for many cancers, they have remained remarkably poor and ambiguous for PDAC. Transabdominal and intraductal ultrasound; endoscopic cholangiopancreatography; contrast-enhanced CT; T_1_, T_2_ and diffusion-weighted MRI; and MR cholangiopancreatography (MRCP), are all regularly used in assessing this disease—but they all find themselves lacking in terms of early detection. [4–7] ^18^F-FDG-based PET is also regularly used, but foremost to reveal metastases. All these imaging modalities also suffer from potential confounding factors, including benign lesions, inflammations and cysts. Coupled to this is PDAC’s dense stroma, decreasing further the sensitivity of most imaging modalities Also problematic is a hypovascularity that often leads to poor tumor perfusion and low uptake of contrast agents. All this endows imaging methods with low sensitivity and poor specificity –especially when trying to detect small (< 2 cm) lesions. Consequently, about 80% of PDAC cases are unambiguously diagnosed in a stage at which the cancer is already metastatic and no longer resectable; nearly an equivalent proportion of these diagnosed patients (~ 80%) will die within one year of diagnosis.

It follows from all this that there is a pressing need for a method to detect PDAC at earlier, more treatable stages. There is also a need of methods that can better assess PDAC treatment effectiveness. Given that PDAC is a profoundly glycolytic cancer, it can be expected to produce abundant lactate; [8] imaging the fluxes involved in this metabolic transformation could, therefore, be a promising method to achieve a specific PDAC mapping. Recent studies based on metabolic imaging of deuterium (^2^H) and carbon-13 (^13^C) support this hypothesis. [9–19] Both thermally and hyperpolarized ^13^C magnetic resonance spectroscopic imaging (MRSI) techniques have been explored in preclinical trials, using glucose and/or pyruvate as the administered glycolytic substrates; in both cases, the MRSI focused on measuring and, when possible, quantifying, the generation of ^13^C-labeled lactate via glycolysis. Pyruvate-based hyperpolarized ^13^C MRSI experiments have also given promising results in small human patient cohorts—even if PDAC’s hypovascularity and the relatively high interstitial fluid pressure of pancreases in general, conspire against the limited observational time scales allotted by nuclear hyperpolarization.

The present work focuses on ^2^H MRSI, an alternative minimally invasive approach, for achieving some of these goals. ^2^H MRSI has already been applied to orthotopic PDAC mouse models. In its most common implementation, tumors were then studied via Deuterium Metabolic Imaging (DMI) experiments, which followed the metabolism of ^2^H_6,6’_-glucose after the latter has been administered by oral, intraperitoneal (IP), or intravenous (IV) routes. [20–33]. When considering organs other than the brain, a ^2^H_3,3’_-lactate peak will then rise above the background noise (or sometimes above a weak natural abundance fat signal) at about 1.2 ppm—but only if generated by the active glycolysis of a tumor. We have investigated DMI’s potential for detecting and mapping PDAC in such a way using a variety of preclinical pancreatic cancer models, and have consistently observed a strong deuterated lactate signature associated with the tumors. Using customized pulse sequences and processing pipelines at high fields, we have thus been able to map pancreatic tumors and their metastases when under 2 mm in diameter, as revealed by lactate concentrations that can be detected ca. 30–60 min after the glucose’s administration, even when in the 0.2–0.3 mM range. In addition, DMI approaches could successfully differentiate tumors from common confounding factors such as pancreatitis [18].

Besides helping reveal the presence of tumors, one of the promises carried by metabolic imaging techniques lies in their ability to monitor alterations in cellular metabolism upon treatment, before morphological changes become apparent. Hyperpolarized ^13^C MRSI has enabled the visualization of such metabolic flux changes upon treatment for key pathways—including glycolysis and the TCA cycle [34–36]. Likewise, it has been proposed that DMI can provide a snapshot of metabolic status without the time constraints imposed by decaying hyperpolarizations nor the radiation burden of PET [37, 38]. A recent study based on imaging the metabolic HDO production revealed a dependence of the label uptake in the tumor on the presence or absence of therapies [39]. The present work explores DMI’s potential to diagnose treatment on a PDAC model, from the standpoint of the glucose uptake and glucose-to-lactate conversion. To this end, animals implanted with a well characterized pancreatic cancer model were divided into treated and untreated cohorts, with the former subject to cyclophosphamide (CP)—a common therapeutic that works either by alkylation of the cancerous cells’ DNA or by modulating the immune system [40, 41]. A clear difference in the rate of tumor growth and the survivability of the two cohorts arose, which was also reflected in the amounts of deuterated lactate generated by the tumors in the presence and absence of treatment. These differences were analyzed upon normalization by tumor volumes, and they revealed correlations between the metabolic kinetics arising from the DMI data and the development of untreated tumors. Particularly notable were correlations between the rate of growth of a particular tumor and both the glucose uptake and the glucose-to-lactate conversion rate. Remarkably, these correlations lost statistical significance upon CP treatment—something which could be useful for prognosing the outcome of this or other given treatments. Observables deriving from the breaking of such correlations that could help diagnose the aggressiveness and/or treatment success of the cancer before visible changes affect a tumor’s structure, are briefly discussed.

## Methods

All experiments were approved by the Weizmann Institute Institutional Animal Care and Use Committee (IACUC), which is accredited by the AAALAC, the US NIH Office of Laboratory Animal Welfare, and the Israel Ministry of Health. Fifteen C57 black mice were implanted with KPC rodent pancreatic ductal adenocarcinoma (PDAC); *n* = 9 of them were subject to chemotherapeutic treatment, and *n* = 6 were used as control.

### Tumor implantation

The KPC cancer mouse model used here has been genetically engineered to present genetic similarities with human PDACs, including common immune defects, barriers to effector cell infiltration, and similar immune checkpoint signaling [42]. The specific KPC-Luc-mCherry line used in this work was obtained from Prof. David Tuveson (Cold Spring Harbor Laboratory). To proceed with the implantation, mice were anesthetized with ketamine/xylazine and given analgesic agents (s.c. 0.05 mg/kg of buprenorphine). A laparotomy (5–10 mm) was performed over the left upper quadrant of their abdomen to expose the peritoneal cavity. The pancreas was carefully brought out onto a sterile field, and KPC tumor cells (2 × 10^4^ cells suspended in 20 ml of sterile PBS mixed with 20 ml Matrigel^®^) were injected into it using a 30-gauge needle. Successful injection was confirmed by the formation of a liquid bleb at the injection site. The pancreas was then gently returned to the peritoneal cavity, and the peritoneum and skin were closed using sutures and autoclips, respectively.

### Cyclophosphamide treatment

This study explored the effects of cyclophosphamide, a common chemotherapeutic used to treat a variety of solid cancers [40, 41]. In the present case, CP treatment started 12 ± 1 days after tumor implantation; this first treatment was followed for each animal by a second, third, etc. treatments, always spaced 7 days apart. The number of treatments given depended on the rate of tumor growth; in all cases, a 10-mm diameter cancer or poor health conditions (whatever set in first), were set as humane end points for the experiments. For each of these weekly treatments, 200 mg of cyclophosphamide per kg of animal body weight were diluted in 25 µL of Dulbecco’s Phosphate Buffered Saline (PBS), and intraperitoneally administered. In the control animals, no cyclophosphamide was injected.

### In vivo MRI procedures

**Fig. 1:**
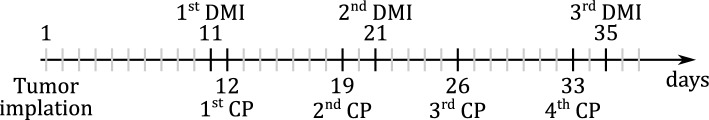
Summary of the experimental timeline, taking 1 as the tumor implantation day. DMI represents the approximate post-implantation days of the ^2^H MRSI scan, and CP the same for the cyclophosphamide treatment. The study refers to scans as occurring 1.5, 3, 5, etc. weeks after implantation; the exact dates for each animal are presented in Table 1

All mice were scanned 11 ± 2 days after tumor implantation; in all cases this first DMI scan happened before the first chemotherapy treatment, and hence it was done on untreated animals for all subjects. The second DMI scans happened for all animals—treated and untreated—10 ± 3 days after the first one; by that time some of the control (untreated) animals already presented suffering behavior, and had been sacrificed. Treated animals showed much slower tumor progression, and hence some of them could be subject to a third and for one animal even a fourth DMI scan—all of these separated by ca. 14 ± 1 days. Figure 1 presents a summary of the experimental timeline that was used, while Table 1 outlines the exact timing of the treatments and of the ^1^H/^2^H MR measurements for each animal.

**Table 1:** Timeline of events underwent by each animal, described in days following tumor implantation. Animals noted “T” were treated with CP; those noted “C” were used as control. Also noted is when animals were euthanized due to health conditions

Week	#1.5		#2	#3		#4	#5
Subject/Procedure	DMI	CP	CP	DMI	CP	CP	DMI
Treatment Group							
T1	8^b^	11	17	18	25	32^a^	-
T2	9	11	17	18	25	32	39^a,b^
T3	10	12	18	19	25	32^a^	-
T4	11^b^	12	18	19^b^	25	32^a^	-
T5	11^b^	13	19	20	25	32	39^,a,b^
T6	12	13	20	21	27^a^	-	-
T7	12	13	20	21	27	34	35^c^
T8	12	13	20	22^b^	27	34	35^a^
T9	12	13	20	22	27	34	35^c^
Control Group							
C1	10	✗	✗	21	29^a^	-	-
C2	10	✗	✗	21^a^	-	-	-
C3	10	✗	✗	21	28^a^	-	-
C4	10	✗	✗	22	29^a^	-	-
C5	11	✗	20^a^	-	-	-	-
C6	12	✗	✗	23	27^a^	-	-

✗—no CP injection (control animals)

^a^Euthanized. Mouse sacrificed because of health condition

^b^Proton imaging only

^c^Mice were sacrificed after the DMI experiment

In preparation for the MRI scans, mice were anesthetized with a 20/80 O_2_/N_2_ stream containing an additional 3% isoflurane to induce sedation. Isoflurane was then taken to ~ 1–2% to keep sedation during the MRI scan. For the DMI experiments, 60 mg of deuterated glucose were diluted in ca. 250 µL of PBS (~ 3 g of glucose per kg of animal body weight) and the ensuing solutions were administered as single boluses. Enriched [6,6’-^2^H_2_]-glucose was purchased from Cortecnet, Voisins-le-Bretonneux, France. The glucose administration was performed via a tail-vein (IV) line injection.

Animals in both groups underwent periodic ^1^H and ^2^H MRI/MRSI until the humane endpoints whereby tumors reached a diameter of 10 mm, suffering behavior was evident, or 20% body weight loss had occurred.

### MR imaging details

The ^2^H/^1^H measurements were conducted on a 15.2T Bruker scanner running Paravision 6, using a 20-mm diameter surface coil manufactured by Varian and tuned to 99.77 MHz (^2^H) and a 40 mm butterfly coil from Bruker tuned to 649.93 MHz (^1^H). An optimized slice-selective, 2D phase-encoded CSI-SSFP sequence was used for the DMI scans ([18], available at https://www.weizmann.ac.il/chembiophys/Frydman_group/software). These SSFP scans used the following parameters: 2 ppm carrier frequency, TR = 11.48 ms, flip angle = 60°, 32 × 32 matrices, in-plane FOV = 40 × 40 mm^2^, 20 mm slices accommodating most of the abdomen excited using 0.63 ms long pulses, a 46-points gradient-free FID sampled at 5 kHz (out of which the 4 initial points had to be discarded as they were corrupted by the digital filtering), weighted signal averaging of the phase-encoding domains, 0.2 ms long phase-encoded gradient pulses, 7 repetitions (for regular average) with number of averages NA = 16 (weighted average and Hamming window). 24–28 ^2^H MRSI images (~ 6 min per image + 1 min for FID control) were acquired, leading to 168–196 min of total experiment time. DMI images were reconstructed by zero-filling the k-space data to 64 × 64 points and applying a 2D Fourier transform; no additional filtering/weighting was applied. The metabolic maps were obtained from such images using the IDEAL method with a priori known chemical shifts (4.7, 3.6, and 1.2 ppm for water, glucose, and lactate, respectively), as described in [17]. (Although reference [17] presents two additional methods to improve the IDEAL approach by incorporating the kinetic dimension, the SNR of the DMI data was sufficient to use only the regular IDEAL method, after incorporating ^1^H-based B_0_ field inhomogeneity maps). To improve field homogeneity, third-order shims were applied using Bruker’s automated shimming procedure, which uses a gradient-echo–based B₀ field map and iterative correction. In addition to these ^2^H acquisitions, ^1^H coronal images were collected using TurboRARE: 10 slices, 0.8 mm thickness, the same in-plane FOV as in the ^2^H MRSI experiments, and a 512 × 512 encoding matrix. Tumor volumes were calculated from the ^1^H anatomical images by manually defining regions of interes (ROIs) on all slices containing the tumor, counting the number of voxels within these ROIs, and multiplying these results by the voxel volume (as given by the voxel width × height × thickness).

To kinetically analyze the information contained in DMI, voxel intensities were converted into absolute concentrations by assuming that the average of the top 10% of preinjection ^2^H-water signal intensities, reflected a 10-mM natural abundance concentration. The SSFP acquisition parameters, as well as the T_1_ and T_2_ values for each species, were taken into account as described in the Supporting Information of [14]. The in vivo T_1_ and T_2_ values were obtained from the Supporting Information of [21]. Metabolic concentrations within these ROIs were extracted from the DMI data by upsampling these 64 × 64 maps using a Kronecker product with an 8 × 8 matrix, to obtain a matrix matching the 512 × 512 size of the ^1^H anatomical images. This ensured that the ROIs manually defined on the ^1^H anatomical images corresponded directly to the same voxels on the upsampled metabolic maps. Postinjection time-evolution concentration curves for HDO, glucose, and lactate for individual ROIs were thereafter found by summing the ^2^H MRI voxel intensities within these regions (in mM), and then normalizing by the number of voxels in each ROI.

### Statistical analyses

Statistical analyses were performed in Python using the “pandas” and “scipy.stats” libraries. To assess the relationships between the estimated variables, Pearson correlation coefficients were calculated for three distinct datasets: treated group, control group, and a combined dataset including all samples. For each of these correlation tables, corresponding p-value tables were also generated. A significance level of *p* < 0.05 was used to determine the statistical significance of each correlation (see text for further details).

### Kinetic modeling

**Fig. 2:**
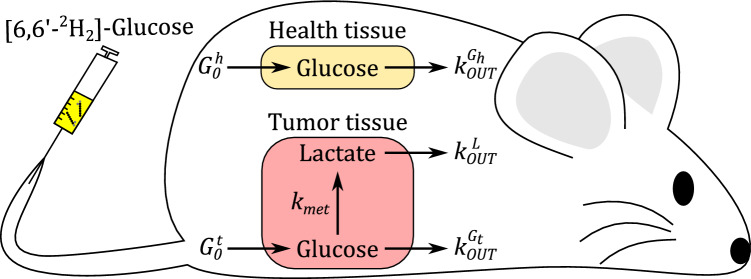
Simplified kinetic model used in this study, assuming simple zero-order kinetics as given by the indicated parameters and disregarding the generation of the HDO (which would have to be considered independently from the glucose-to-lactate conversion process anyhow)

To analyze the information that DMI’s metabolic maps and their time courses carry, the data were analyzed based on the kinetic model in Fig. 2 –that is a simplified version of the models described in [43, 44]. This model disregarded the exchange process between the deuterated glucose in healthy tissues (G_h_, exemplified by the kidney) and in the tumor (G_t_); this was done as no evidence for such an exchange became available from glucose signals that were maximal at the first available time point for both kind of tissues. Also, for the sake of simplicity, the generation of metabolic water from glucose was not included in the analysis. Data interpretation rather focused on the glucose-to-lactate kinetics, as defined by the three differential equations.

$$\frac{d{G}_{h}}{dt}={-{k}_{out}^{{G}_{h}}\bullet G}_{h}$$

$$\frac{d{G}_{t}}{dt}={-{k}_{out}^{{G}_{t}}\bullet G}_{t}$$

$$\frac{dL}{dt}=-{k}_{out}^{L}\bullet L+ {{k}_{met}\bullet G}_{t}$$

where *G*_*h*_*, G*_*t*_ and *L* are the time-dependent intensities observed in the DMI experiments for glucose in the kidney, glucose in the tumor and lactate in the tumor, respectively; ${k}_{out}^{X}$ are constants describing the outflow/consumption of the respective metabolites in their environs, and ${k}_{met}$ is the metabolic rate of lactate generation from the glucose present in the tumor. The solutions to these equations are

4 $${G}_{h}(t)={G}_{0}^{h}\bullet {e}^{-{k}_{out }^{{G}_{h}}\bullet t}$$

5 $${G}_{t}\left(t\right)={G}_{0}^{t}\bullet {e}^{-{k}_{out }^{{G}_{t}}\bullet t}$$

6 $$L\left(t\right)={G}_{0}^{t}\bullet {k}_{met}\bullet \frac{{e}^{{-k}_{out }^{L}\bullet t}-{e}^{-{k}_{out }^{{G}_{t}}\bullet t}}{{k}_{out}^{{G}_{t}}-{k}_{out}^{L}}$$

These expressions were used to fit the experimental time traces according to initial conditions that included *L*_*t*_(0) = 0 and glucose intensities ${G}_{h}\left(0\right)={G}_{0}^{h}$ at the kidney and ${G}_{t}\left(0\right)={G}_{0}^{t}$ at the tumor. These fits of ${G}_{h}(t)$, ${G}_{t}\left(t\right), L(t)$ focused on the spatially averaged values extracted from the voxels covering these organs for these three observables. In all instances, Eqs. (4)–(6) could closely reproduce the kinetics of the various data sets; all such fits are presented for completion in the Supporting Information Section S3.

## Results

**Fig. 3:**
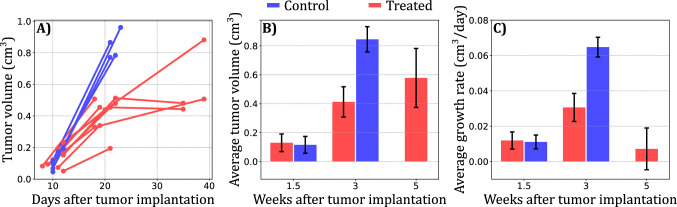
**A** Tumor sizes measured for the full set of studied animals as a function of days elapsed from the cancer implantation; note that none of the animals had been treated in the first set of measurements (point at ca. 11 ± 2 days). **B** Same as (**A**) but pooling together animals according to treatment cohort and to approximate number of weeks elapsed since tumor implantation. **C** Same as (**B**) but describing the rate of tumor growth, as given by the change in tumor size divided by the days elapsed in-between the evaluation of the size by MRI. All measurements arising from ^1^H TurboRARE images collected at 15.2T

As mentioned, two cohorts of PDAC-implanted animals were studied: one received CP chemotherapy, while the other served as an untreated control. Table 1 summarizes the precise timing of treatments and ^1^H/^2^H MR measurements for each subject; overall this followed the schedule in Fig. 1, with minor deviations imparted due to the scanner’s availability. As can be seen from the Table, the animals exhibited significantly prolonged lifetimes upon treatment. This arose from substantially slower rates of tumor growth upon CP administration: Fig. 3 plots how tumor volumes and tumor growth rates, both as measured via high-resolution ^1^H MRI, changed for each group of animals as a function of the days/weeks following the cancer cells implantation. No significant differences in size are noticed between the tumors of the two batches up to day 11 (week “1.5”), which was prior to the point at which the first CP treatment happened. At this stage there is a bifurcation in the rates of tumor growth, with treated animals exhibiting slower tumor growth and extended survivals. Table S1 in Supporting Information Section 1 summarizes the tumor volumes measured for all animals throughout their lifetimes via ^1^H MRI.

**Fig. 4:**
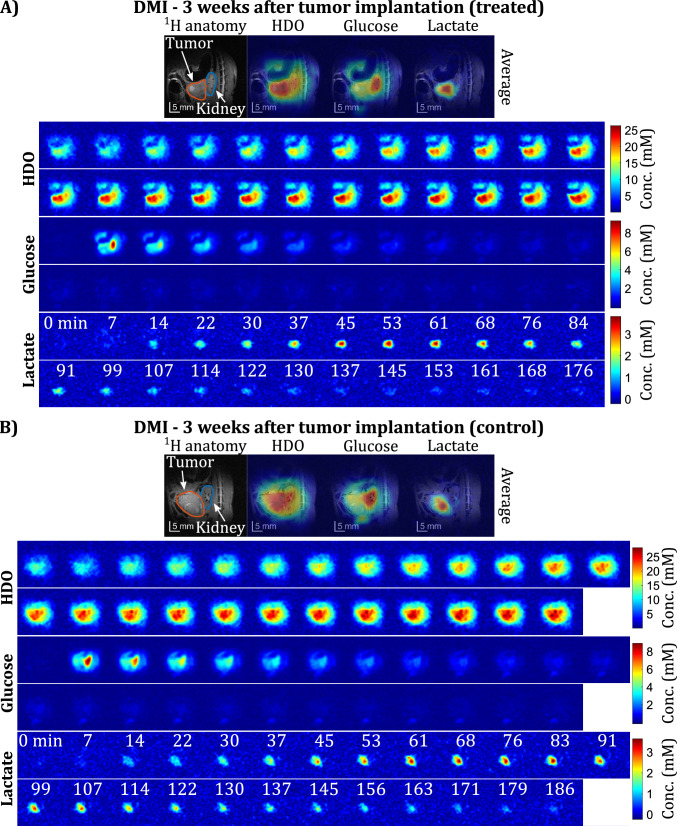
^2^H images collected over time following the injection of deuterated glucose, acquired 3 weeks after tumor implantation in a treated and a control subject. Each panel represents a time point in the kinetic acquisition, spaced approximately 7 min apart, and covering a total duration of ~ 180 min. Summed images of deuterated glucose, water, and lactate over the full scan are overlaid on anatomical ^1^H images, highlighting the tumor and kidney of each animal. See the Supporting Information for a description of all data for all animals examined in this study

In parallel with these ^1^H MRI size assessments, DMI measurements were performed on both the treated and control animals, as per the schedule in Table 1. Figure 4 presents representative DMI results for untreated and treated animals, acquired in both cases 3 weeks after tumor implantation. In both instances the three top rows show single-slice images resolved by the CSI-SSFP sequence used in these experiments for the ^2^H-water, ^2^H_6,6’_-glucose, and ^2^H_3,3’_-lactate peaks. Images are here shown as a function of time elapsed since the ^2^H_6,6’_-glucose bolus injection, collected at ca. 7 min spacings; no background (fat) suppression had to be used. Shown underneath these time series are sums of the images collected over the total scanning time for the deuterated glucose, water, and lactate; these are overlaid over the corresponding anatomical ^1^H images, highlighting the tumor and a kidney that was here used as an example of “healthy” tissue. Also clear in some glucose images is the bladder, which could show an overspill of this metabolite into urine reflecting either a relatively high administered amount of sugar and/or an impaired pancreatic function. Supporting Information Section 2 shows the remaining, full set of all the DMI results in this study.

**Fig. 5:**
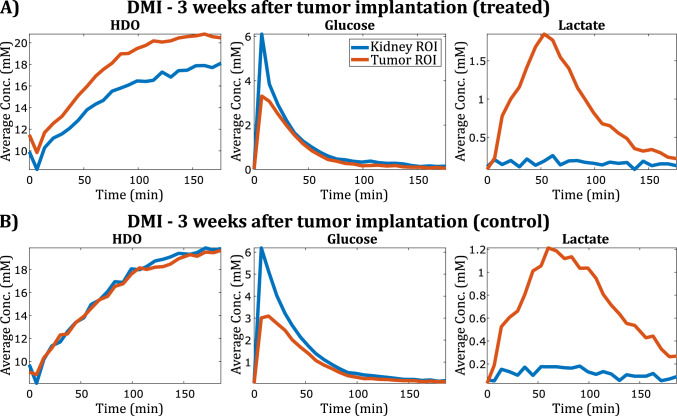
Average metabolic concentration curves derived from the metabolic maps for the tumor and kidney ROIs, as defined in the corresponding ^1^H anatomical images in Fig. 3. We ascribe the slight dip that is sometimes seen in the HDO intensity shortly after the glucose injection to motion-derived interferences in the SSFP-CSI reconstruction

The metabolic kinetics for all three sets of time-resolved images introduced in Fig. 4 for control and treated cases are shown in Fig. 5, which shows the ROI-averaged concentrations arising from the kidney and tumor regions, plotted as a function of post-injection time. The behavior observed for both kinds of animals is similar. In both treated and untreated batches, the highest ^2^H_6,6’_-glucose signal is observed in the first data point in both the kidney and the tumor; in nearly all animals, this initial amount of glucose is usually higher in the kidneys. From this maximum onwards, the glucose decays in both organs within ca. 2–3 h, with rates that are usually higher for the kidney. ^2^H-water, by contrast, steadily rises throughout the course of the 2+ -hour scan, with a slight positive concentration bias in the tumor location. Lactate production, on the other hand, is markedly different in the tumor and the kidney: it is only observed in the former, reaching maximal concentrations ca. an order of magnitude smaller than those of the glucose ≈60–90 min after injection, and eventually washing out. The ^2^H-water signal tends to plateau at ca. 180 min; about at the same time as the deuterated lactate finally goes back to zero. At this point, it is likely that all the deuterium that was administered with the ^2^H_6,6’_-glucose—some of which may have become invisible due to conversion into deuterated glycogen—has been consumed.

**Fig. 6:**
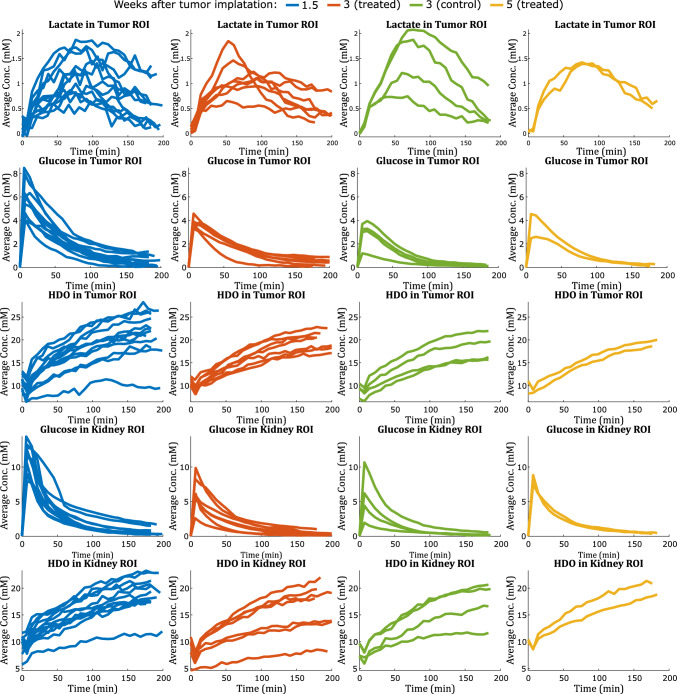
Time dependencies of the average concentrations measured by ^2^H MRSI for all the animals and scans in these studies in the kidney and tumor regions, as a function of time elapsed since the deuterated glucose administration

Figure 6 presents collective results observed in this study, by plotting for each of the metabolites, for ROIs encompassing the tumors and healthy tissue (kidney), and for different treatment conditions, the DMI kinetics observed as a function of time postinjection. These curves are shown for all the animals in the study, and give an idea of systematic differences with and without treatment, as well as of the scattering within each cohort.

**Fig. 7:**
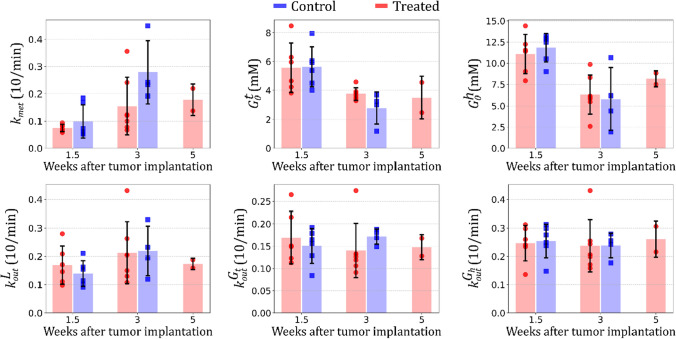
Kinetic and glucose uptake parameters arising from the fitting of the DMI data, for the full set of studied animals as a function of weeks elapsed from the cancer implantation. These graphs pool together animals according to treated/control cohorts as well as approximate number of weeks elapsed since tumor implantation (these are approximate week values; Table 1 lists the exact dates for each animal’s scan). Note that the treated group at 1.5 weeks after tumor implantation consists of untreated animals, but is defined here as ‘treated’ to show that the baselines of the two groups are similar

Numerical fits of Eqs. (4)–(6) to the average concentration data obtained from DMI are presented in Supporting Section 3. Supporting Section 4 summarizes in Table S2 the kinetic parameters emerging from such ROI-averaged concentration fits, for the full cohorts of treated and control animals. These parameters include the four kinetic constants ${\{{k}_{out}^{X}\}}_{X=L,{G}_{h},{G}_{t}}$ and ${k}_{met}$, as well as the initial glucose concentrations ${G}_{0}^{h}$, ${G}_{0}^{t}$ arising when fitting the volume-normalized concentrations of glucose and lactate detected by DMI. Figure 7 summarizes the kinetic rates and initial glucose values for the full cohort as a function of the weeks elapsed since the PDAC implantation, while discriminating treated from control animals. Trends that are apparent from these graphs include an increase in the rate of lactate production ${k}_{met}$ with time elapsed post-implantation. Also noticeable is a decrease in glucose uptake *G*_*0*_ both in healthy and tumorous tissues as the disease progresses. The full list of parameters is listed in Table S2 and plotted in Fig. 7 as a function of the time elapsed for each animal as a function of the tumor implantation (see Table 1 for the exact days elapsed since implantation for each animal and study).

**Fig. 8:**
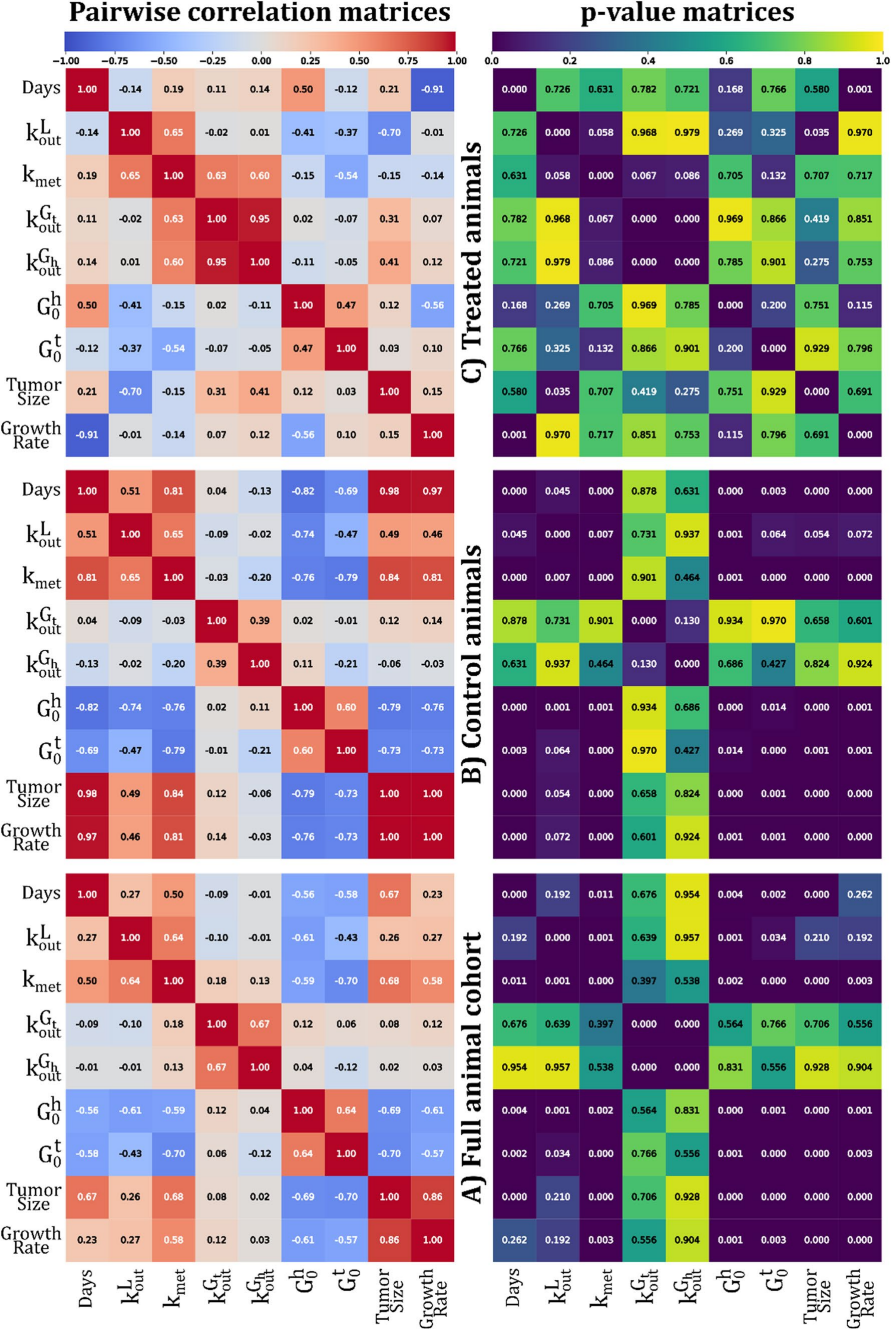
Correlation matrices among the stated observables and corresponding p-values arising upon considering: **A** The full cohort of animals in this study. **B** The untreated animals used as controls. **C** The cohort of treated animals. Kinetic parameters are as defined in Fig. 2; Days refer to the post-implantation days at which the measurements were taken; tumor size and tumor growth rate are as defined in Fig. 3. See text for further details

Figure 8 explores pairwise correlations between the DMI metabolic kinetic parameters and the progress of the disease as revealed by tumor sizes and tumor growth rates—when extracted for each animal individually. These correlations include the various parameters associated with the DMI metabolic fits, as well as: (1) the days elapsed since the tumors’ implantations and the day of the MRI scan, (2) the absolute size of the tumors reached on the day of the MRI scan, and (3) the rate of tumor growth as established by the sizes measured by consecutive MRI scans divided by the days elapsed between scans. Figure 8A presents these data when considering the animal cohort of this study as a whole; while informative, these results may naturally be influenced by the application or the lack of CP treatment for a specific animal. Further insight is presented in Fig. 8B and 8C, which center on the cohorts of control (including all pre-treatment mice) and treated rodents, respectively. For ease of analysis, pale colors in these matrices reflect weak correlations (absolute values ≤ 0.5) while stronger red/blue colors reflect more defined correlations (absolute values ≥ 0.6). Shown next to each of these correlation matrices are the corresponding *p* value matrices, highlighting the significance or absence thereof of the pairwise correlations according to color—with deeper blues reflecting more meaningful, *p* ≤ 10^–3^ correlations.

## Discussion

Figures 7 and 8 summarize the main results of this study. Among all parameters shown in Fig. 7, the increase in ${k}_{met}$ in treated animals compared with control animals is arguably reflective of an increased tumoral activity. This increase continues but appears to slow down by the chemotherapy, as evidenced by the lower increases in the ${k}_{met}$ constant for the final weeks of the treated animals. Another parameter is glucose uptake (*G*_*0*_), which decreases as the disease progresses, probably reflecting an altered metabolism associated to the pancreas’s impairment.

As several uptake concentrations and metabolic rates arise from the DMI exams, pooling them together as in Fig. 7 blurs the message that they could convey individually. Also, correlations against the aggressiveness of a particular tumor or vs the progress of the CP treatment—as could be revealed, for instance, via the rate of tumor growth as introduced in Fig. 3—may also be lost by this pooling of data. It may, therefore, be instructive to interpret all the ensuing parameters through pairwise correlations rather than collectively. Figure 8 treats the data in such manner. Considering the cohort as a whole reveals several strong (± 0.7–± 1 range, *p* < 10^–3^) correlations (Fig. 8A). An even larger number of strong, meaningful correlations appear when considering separately the untreated animals (Fig. 8B). By contrast, most correlations are broken upon CP treatment (Fig. 8C).

Starting with the correlations observed for the untreated cohort: the strongest (> 0.95) and most plausible (*p* < 10^–10^) links arise—as perhaps could have been expected for an aggressive, fast-growing cancer such as PDAC—among the morphological aspects of the tumor growth. Specifically, between the size of the tumor, the rate of tumor growth, and the number of days elapsed since the cancer’s implantation. The next strongest correlation to emerge (> 0.8, *p* ≈ 5^.^10^–5^) links the ${k}_{met}$ rate describing the lactate rate of production from glucose at a tumor, with the growth rate and/or size of the tumor. This suggests that quantifying the rate of metabolic lactate generation could serve as predictor on the rate of growth of a PDAC tumor—and thereby of its aggressiveness. Smaller in absolute value (≈ −0.8) and somewhat less plausible (*p* ≈ 2^.^10^–4^) but still meaningful, are the correlations shown by the capacity of both healthy and cancerous tissues to take up the glucose—reflected by the ${G}_{0}^{h}$, ${G}_{0}^{t}$ derived from the DMI fits for healthy and cancerous tissues, respectively—and the growth rate and/or tumor size. Under normal conditions one could have assumed these uptake parameters to stay approximately constant, reflecting the constant concentrations and injection volumes of deuterated glucose employed for all animal studies. However, the steady decrease of ${G}_{0}^{h}$ and ${G}_{0}^{t}$ as the tumor progresses, probably reflects the impaired capacity of the animals to absorb the injected glucose as the pancreatic cancer advances. Several other meaningful pairwise linkages among all these observables (e.g., among ${G}_{0}^{h}$ and ${k}_{met}$ or among ${G}_{0}^{t}$ and tumor size) follow from these main two metabolic/morphologic correlations.

Remarkably, basically all these strong, clear correlations disappear or lose plausibleness upon subjecting animals to chemotherapy—including basic correlations such as that between tumor_size/growth_rate and days elapsed since the cancer implantation (Fig. 8C). By contrast, a new correlation emerges between the washout rates of the glucose in healthy and cancerous tissues; this, however, may be reflective of side effects—many of which are known to be associated with CP chemotherapy.

In view of the significant effects that the treatment appears to have on the metabolic parameters, we sought whether a single observable reporter could be found that would provide a distinction between a promising or a discouraging outcome for the chemotherapy treatment. Hyperpolarized ^13^C MRSI studies, for instance, have suggested the progression of the lactate-to-pyruvate ratio as a potential treatment reporter [34–36]; it is worth exploring further if a trustworthy DMI reporter could likewise predict tumor aggressiveness and/or the effectiveness of a given treatment. Such search is underway.

## Conclusions

The purposes of this study were twofold: to confirm that a chemotherapeutic treatment or lack thereof would reflect on the metabolic parameters measured by ^2^H MRSI on an aggressive cancer, and to look for a simple DMI-derived reporter that could help discriminate the success or failure of a common chemotherapeutic treatment. To this end we focused on a PDAC mouse model and explored the effects that cyclophosphamide had in its DMI metabolic reporting. Strong links were then observed between the aggressiveness of the tumors, as reported for instance by rates of tumor growth, and basic parameters arising from the DMI data like the normalized metabolic glucose-to-lactate conversion rate *k*_*met*_ at the site of the tumor (positive correlation) or the amount of uptaken glucose at the tumor (negative correlation). These correlations disappeared upon successful treatment, confirming the close association between the rate of tumor growth and a mappable metabolic tumor activity. On the basis of these different behaviors it was ascertained which parameters deriving from the metabolic fits—e.g., the ratio between the rate of glucose-to-lactate conversion and the glucose uptake of the tumor—changed together with the onset of the treatment. This bodes well for the use of these glycolytic markers in the prognosis of the success/failure of a chosen treatment modality. The generality of such findings is currently under further investigation.

## Supplementary Information

Below is the link to the electronic supplementary material.

Supplementary file1 (PDF 8359 KB)** Supplementary Material.** Section "Introduction": Table with volumes estimated for tumors –all animals, all scans. Section "Methods" : Experimental maps arising from the ^2^H MRSI studies for all animals. Section "Results" : Summary of data fits for all experimental ^2^H MRSI (lactate, glucose), for every animal and scan. Section 4: Listing of all fitting parameters emerging from the metabolic studies

## Data Availability

All data needed to evaluate the conclusions in the paper are present in the paper and/or the Supplementary Materials. Additional information is available from the authors upon suitable request.
