# Supplementary material for: Assessing the treatment of pancreatic ductal adenocarcinoma by deuterium metabolic imaging: a preclinical study
Source: MAGMA. 2026 Apr 6;39(3):417–30. doi: 10.1007/s10334-026-01340-z (PMC13354705; doi:10.1007/s10334-026-01340-z)
Supplement: Supplementary file 1 — Supplementary file1 (PDF 8359 KB) Supplementary Material. Section "Introduction": Table with volumes estimated for tumors –all animals, all scans. Section "Methods" : Experimental maps arising from the 2H MRSI studies for all animals. Section "Results" : Summary of data fits for all experimental 2H MRSI (lactate, glucose), for every animal and scan. Section 4: Listing of all fitting parameters emerging from the metabolic studies [file 10334_2026_1340_MOESM1_ESM.pdf]

Supporting Information for

Assessing the treatment of pancreatic ductal adenocarcinoma by deuterium metabolic imaging: A preclinical study

Elton T. Montrazi,[1] Maya Kovalevsky,[1] Keren Sasson,[2] Lilach Agemy,[2] Avigdor Scherz,[2] Lucio Frydman[1,*]

Departments of [1]Chemical and Biological Physics and [2]Plant and Environmental Sciences, Weizmann Institute of Science, Rehovot, Israel

*To whom correspondence should be addressed. E-mail: lucio.frydman@weizmann.ac.il

Section 1: Table with volumes estimated for tumors –all animals, all scans.

Table S1: Volume arising from ^{1}H TurboRARE images collected as function of days elapsed from the cancer implantation. T: treated animal. C: control animal

	Day	Vol.(cm^3)	Day	Vol.(cm^3)	Day	Vol.(cm^3)
T1	8	0.082	18	0.508	-	-
T2	9	0.094	18	0.329	39	0.507
T3	10	0.122	19	0.455	-	-
T4	11	0.073	19	0.339	-	-
T5	11	0.195	20	0.439	39	0.882
T6	12	0.05	21	0.194	-	-
T7	12	0.163	21	0.454	35	0.443
T8	12	0.233	22	0.48	-	-
T9	12	0.152	22	0.513	35	0.481
C1	10	0.118	21	0.866	-	-
C2	10	0.045	-	-	-	-
C3	10	0.067	21	0.771	-	-
C4	10	0.097	22	0.784	-	-
C5	11	0.172	-	-	-	-
C6	12	0.193	23	0.961	-	-

Section 2: Metabolically-resolved experimental maps and kinetically-resolved time series arising from the ^{2}H MRSI on all animals and post-implantation times examined in this study.

The panels in the following 28-pages Figure summarizes experimental maps arising from the ^{2}H MRSI studies for all animals. The present Section, as well as the remaining Sections in the Supporting Information, were assigned a unique identifier to specify their treatment group, subject number, and scan time point. The naming convention used here follows the format XY-DZ, where:

- X denotes the treatment group (T for Treated or C for Control).
- Y represents the subject number within group X(e.g., 1,2,3, etc.).
- DZ indicates the DMI scan number (D1 for the first scan, D2 for the second, and so on).

For example, a subject labeled T9-D3 refers to the ninth subject of the Treated group at their third DMI scan. Likewise, C2-D1 refers to the second animal of the Control group in its first scan. Etc.

Note that due to complications that arose in the tail-vein injection, not all animals had successful DMI scans (which are therefore not reported).

Section 2.1. Treatment Group

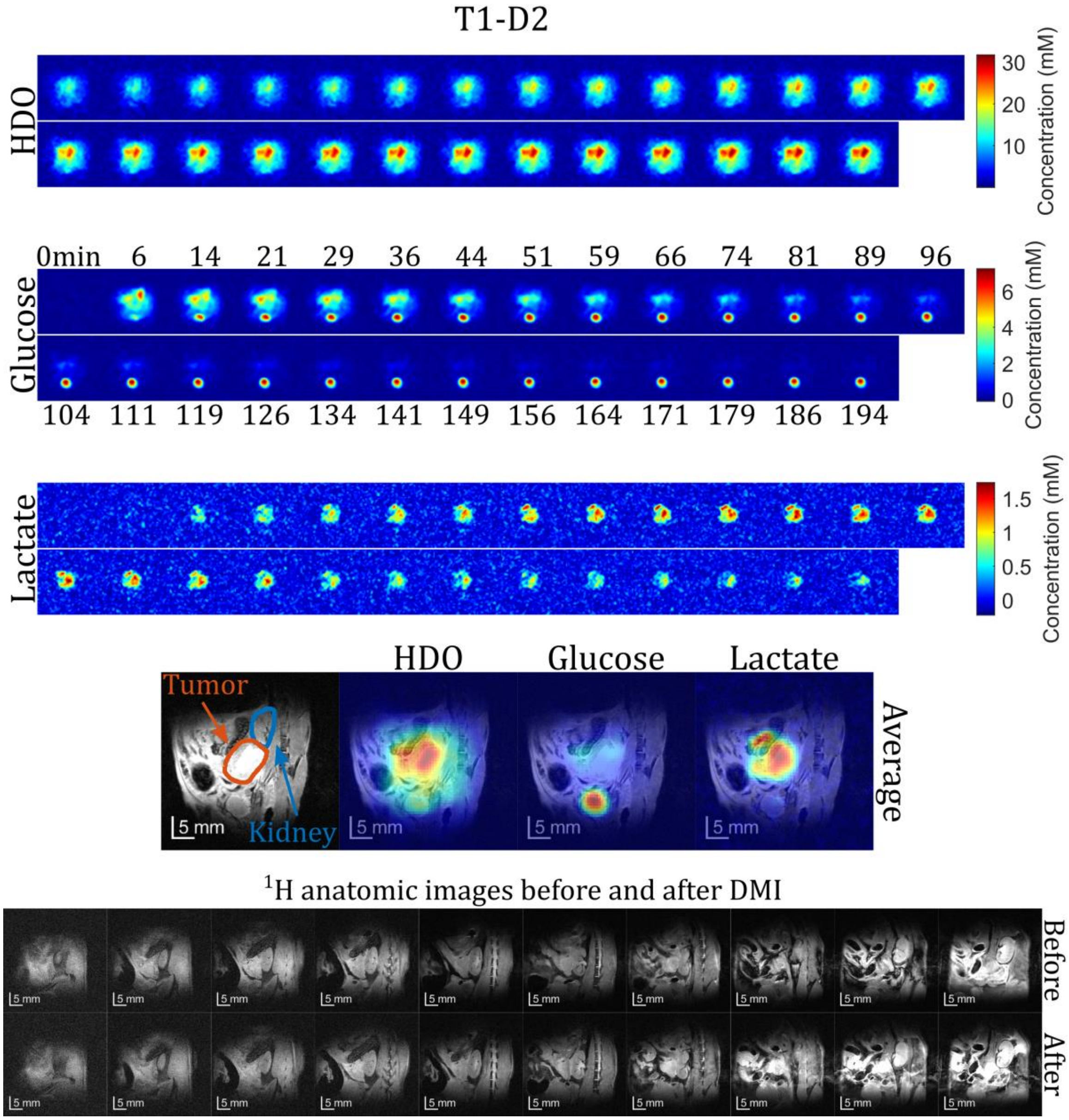

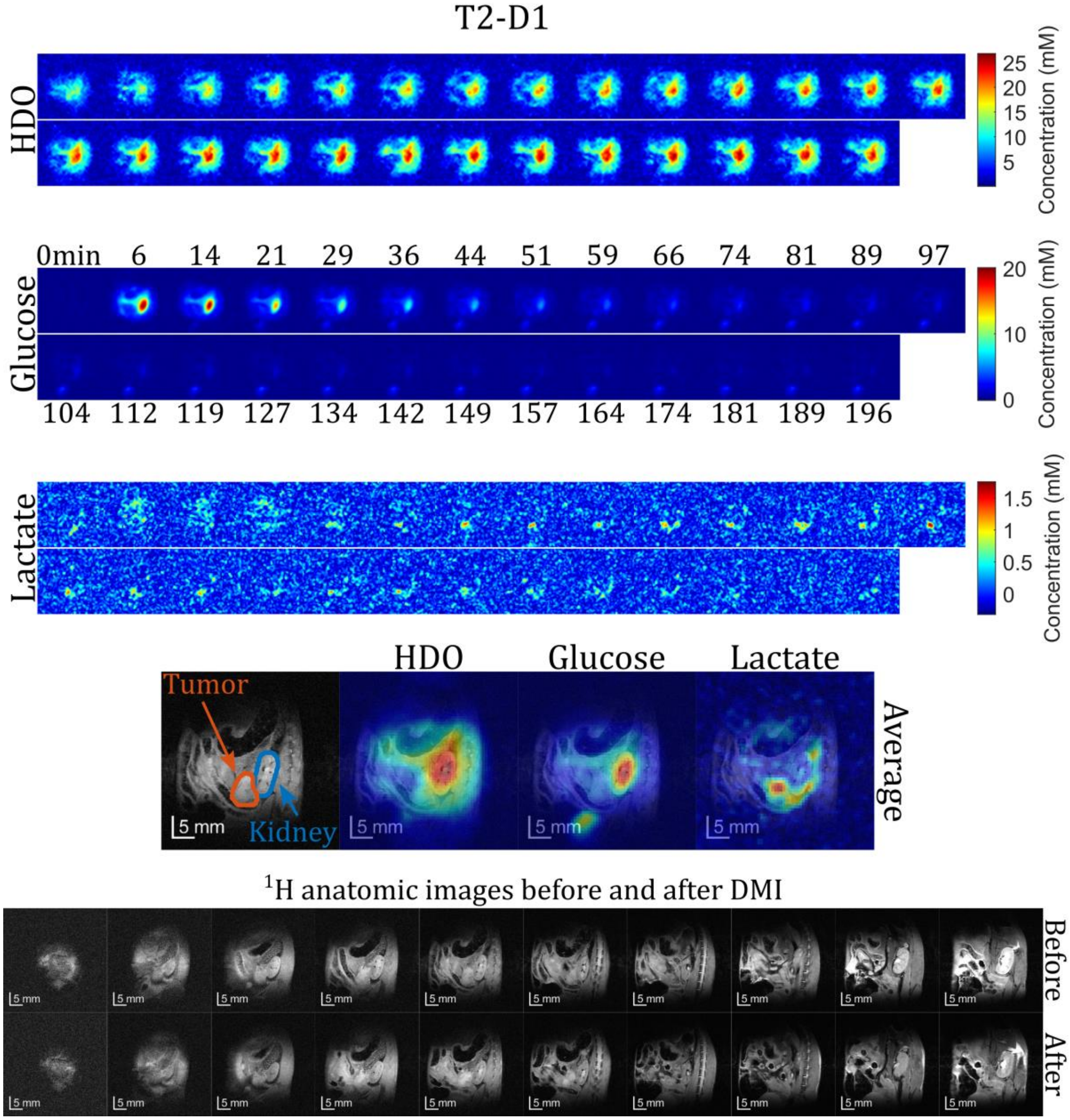

T2-D1
HDO
Concentration (mM)
25
20
15
10
5
0min
6
14
21
29
36
44
51
59
66
74
81
89
97
Glucose
20
10
0
104
112
119
127
134
142
149
157
164
174
181
189
196
Lactate
1.5
1
0.5
0
HDO
Glucose
Lactate
Tumor
Kidney
5 mm
Average
^{1}H anatomic images before and after DMI
Before
After

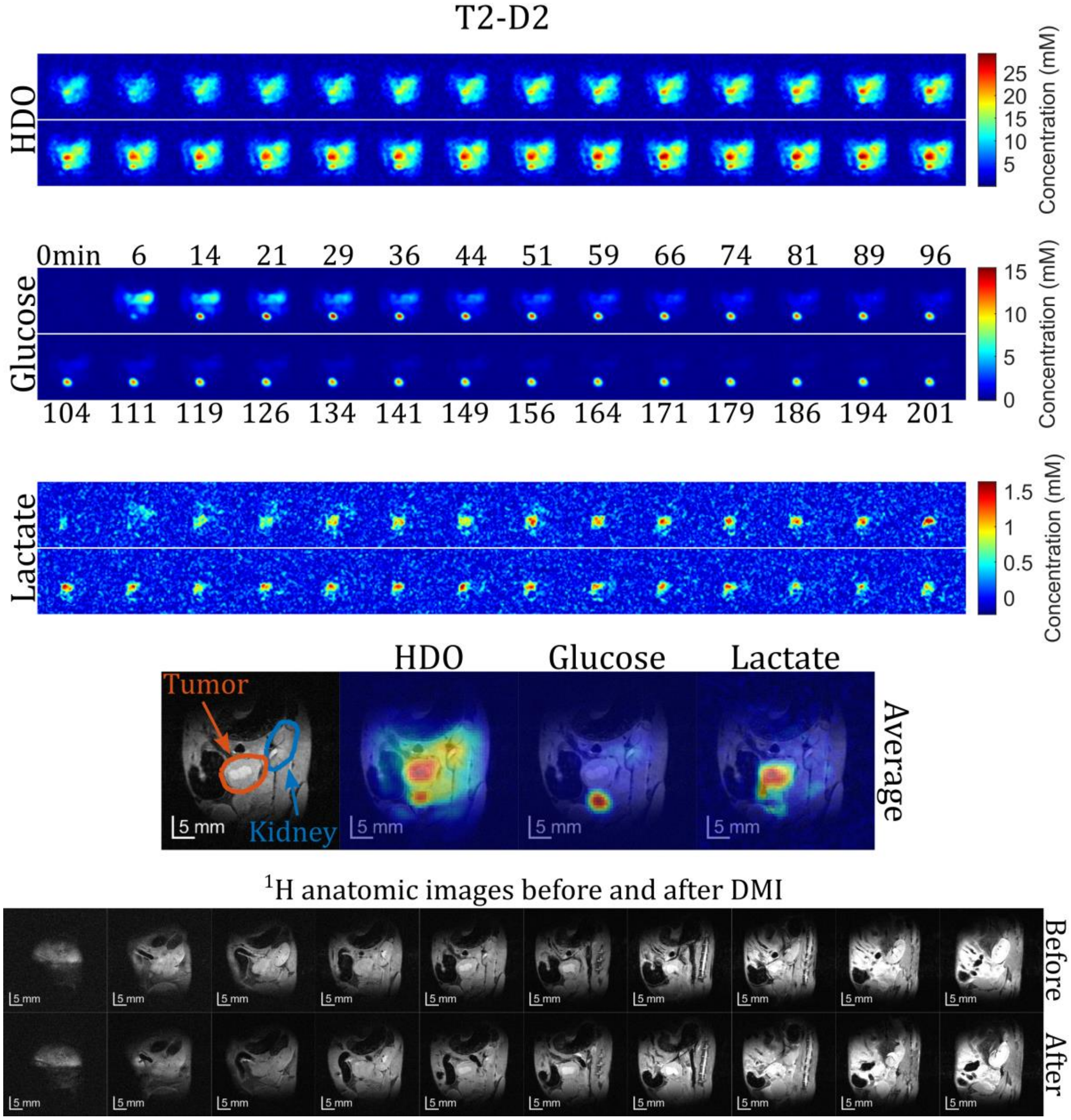
T2-D2
HDO
Concentration (mM)
25
20
15
10
5
0min 6 14 21 29 36 44 51 59 66 74 81 89 96
Glucose
Concentration (mM)
15
10
5
0
104 111 119 126 134 141 149 156 164 171 179 186 194 201
Lactate
Concentration (mM)
1.5
1
0.5
0
HDO
Glucose
Lactate
Tumor
Kidney
5 mm
Average
^{1}H anatomic images before and after DMI
Before
After

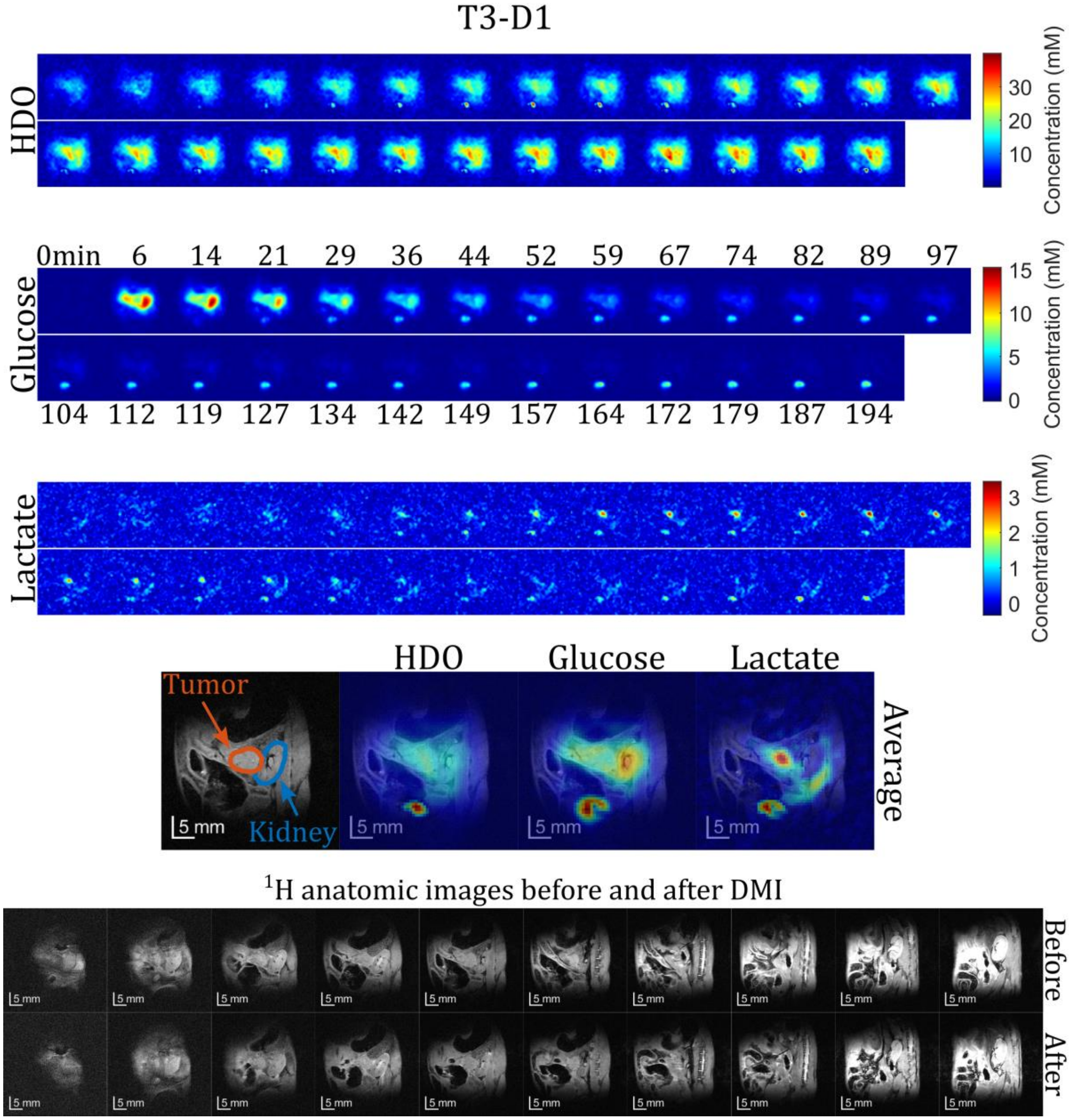
T3-D1
HDO
Concentration (mM)
30
20
10
0min 6 14 21 29 36 44 52 59 67 74 82 89 97
Glucose
15
10
5
0
104 112 119 127 134 142 149 157 164 172 179 187 194
Lactate
3
2
1
0
HDO Glucose Lactate
Tumor
Kidney
5 mm
Average
^{1}H anatomic images before and after DMI
Before
After

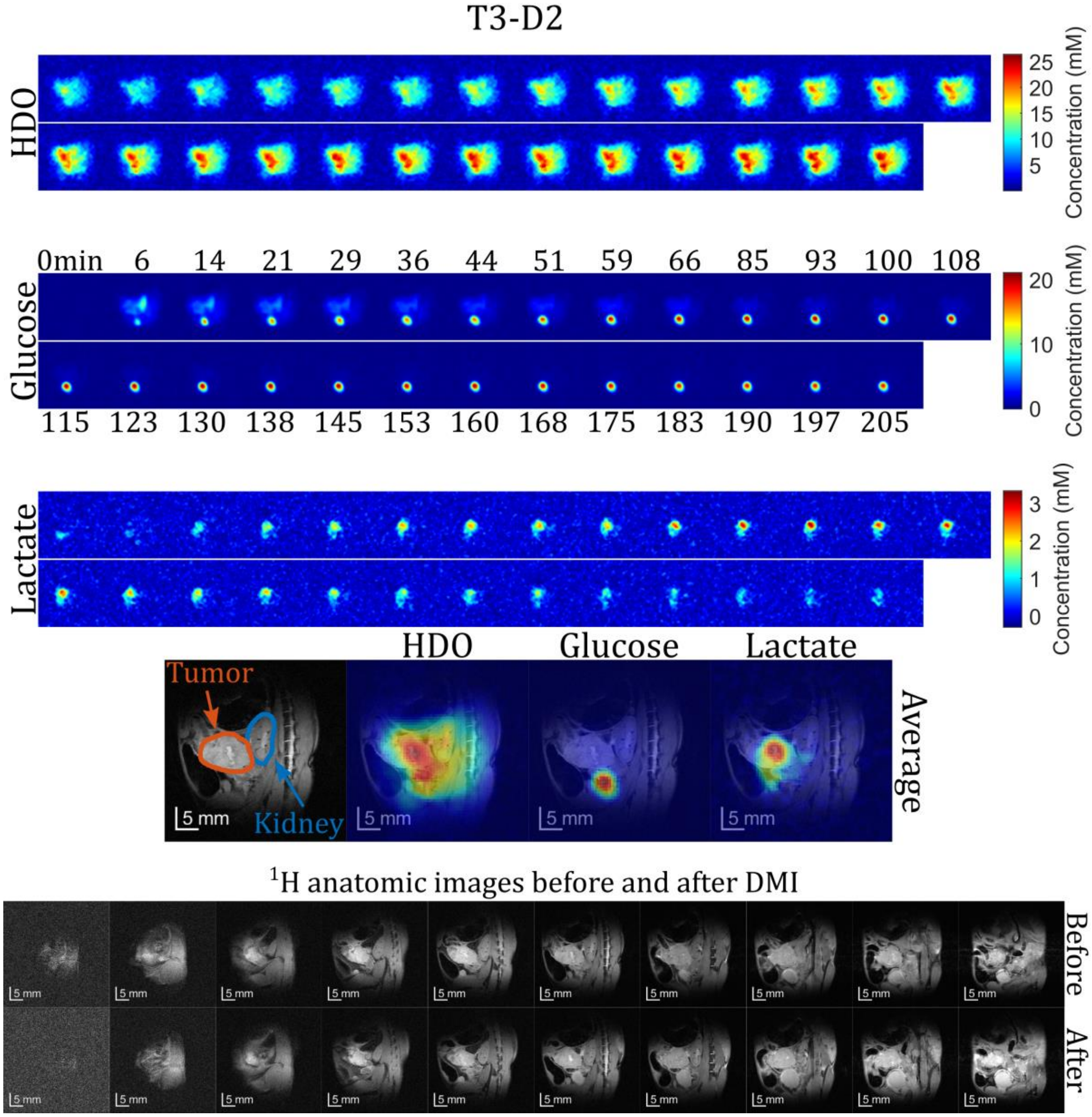
T3-D2
HDO
Concentration (mM)
25
20
15
10
5
0min
6
14
21
29
36
44
51
59
66
85
93
100
108
Glucose
20
10
0
115
123
130
138
145
153
160
168
175
183
190
197
205
Lactate
3
2
1
0
HDO
Glucose
Lactate
Tumor
Kidney
5 mm
Average
^{1}H anatomic images before and after DMI
Before
After

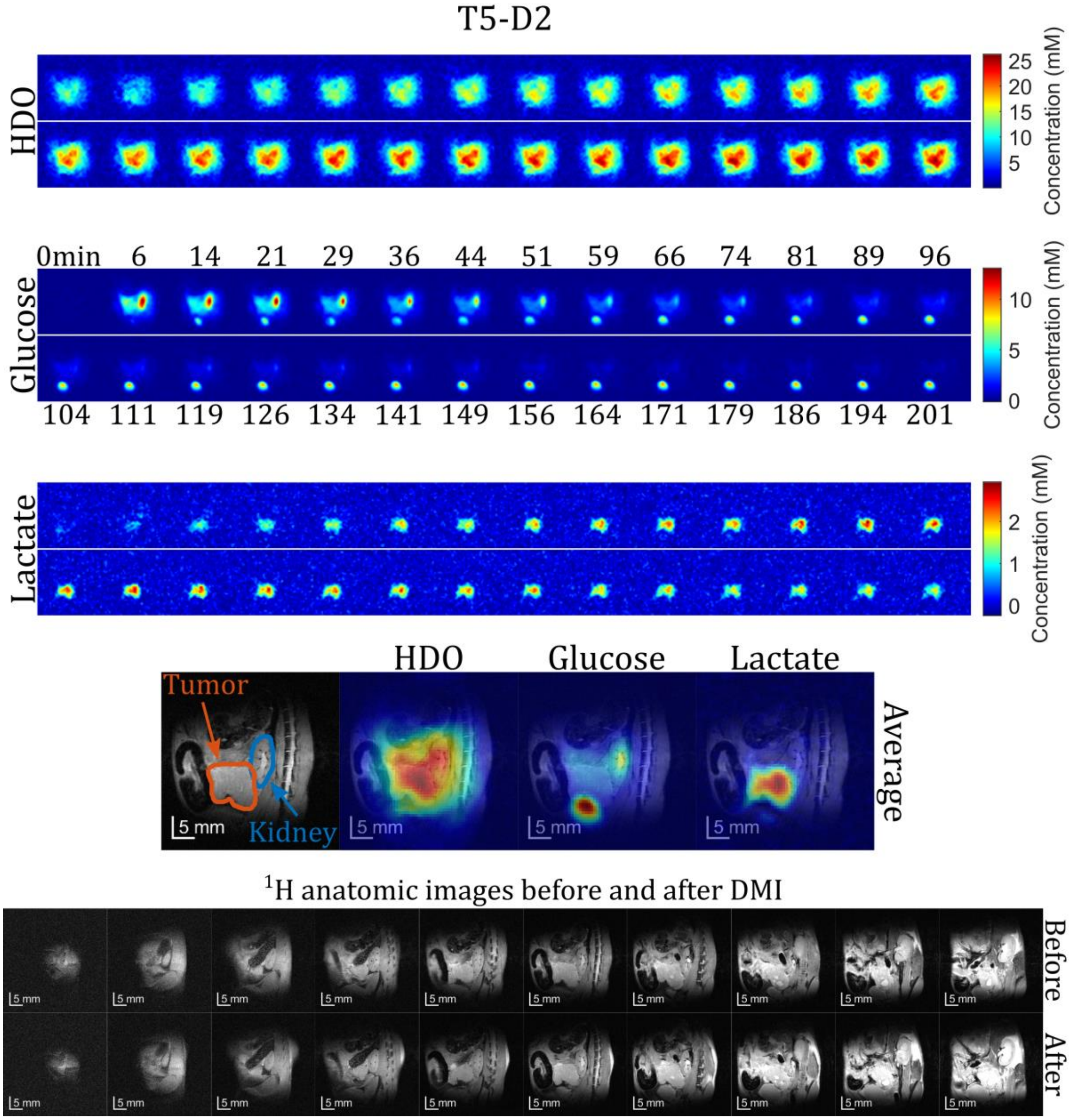
T5-D2
HDO
Concentration (mM)
25
20
15
10
5
0min 6 14 21 29 36 44 51 59 66 74 81 89 96
Glucose
104 111 119 126 134 141 149 156 164 171 179 186 194 201
10
5
0
Lactate
2
1
HDO Glucose Lactate
Tumor
Kidney
5 mm
Average
¹H anatomic images before and after DMI
Before
After

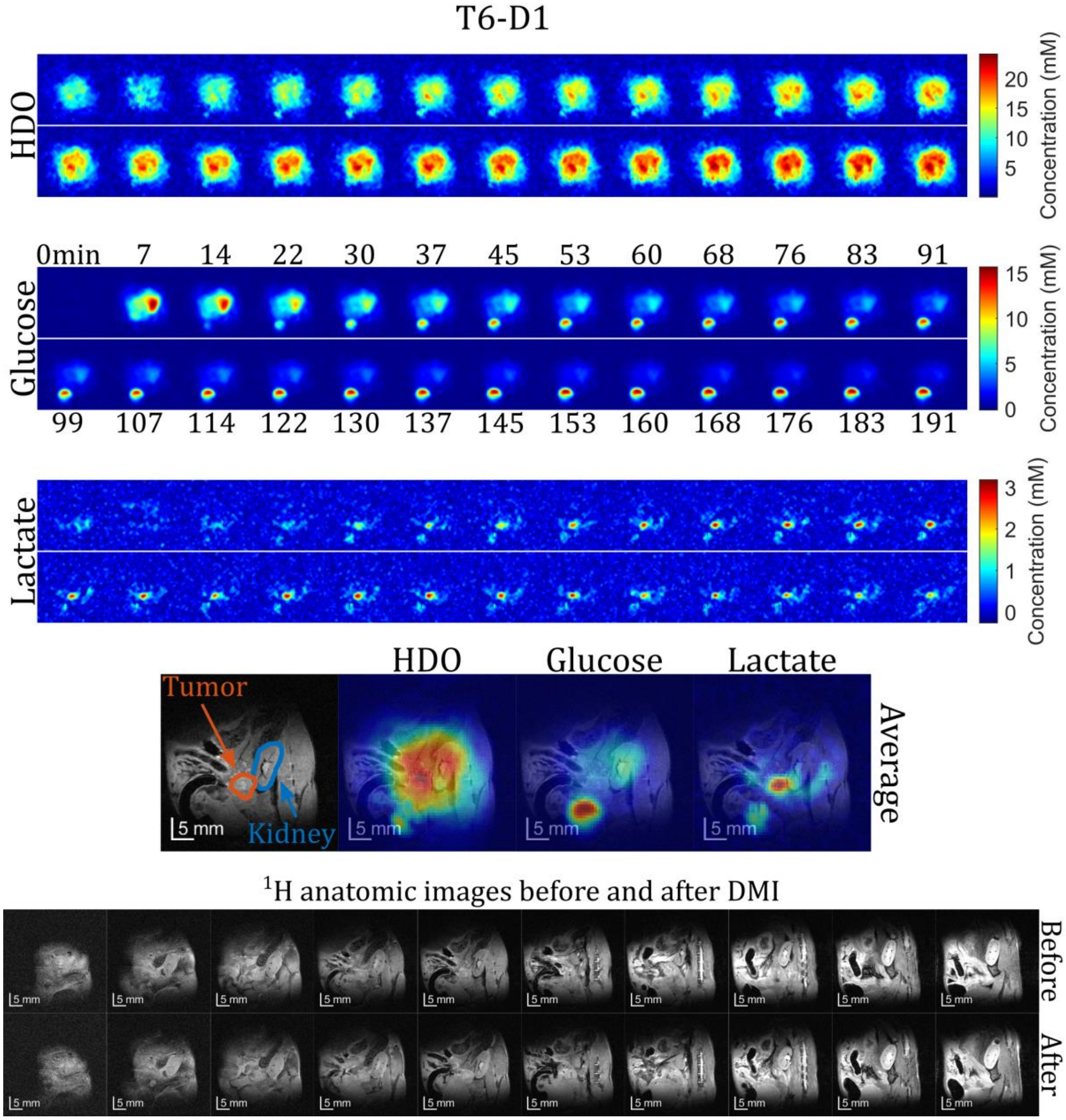
T6-D1
HDO
Glucose
Lactate
0min
7
14
22
30
37
45
53
60
68
76
83
91
99
107
114
122
130
137
145
153
160
168
176
183
191
Concentration (mM)
20
15
10
5
0
3
2
1
HDO
Glucose
Lactate
Tumor
Kidney
5 mm
Average
1H anatomic images before and after DMI
Before
After

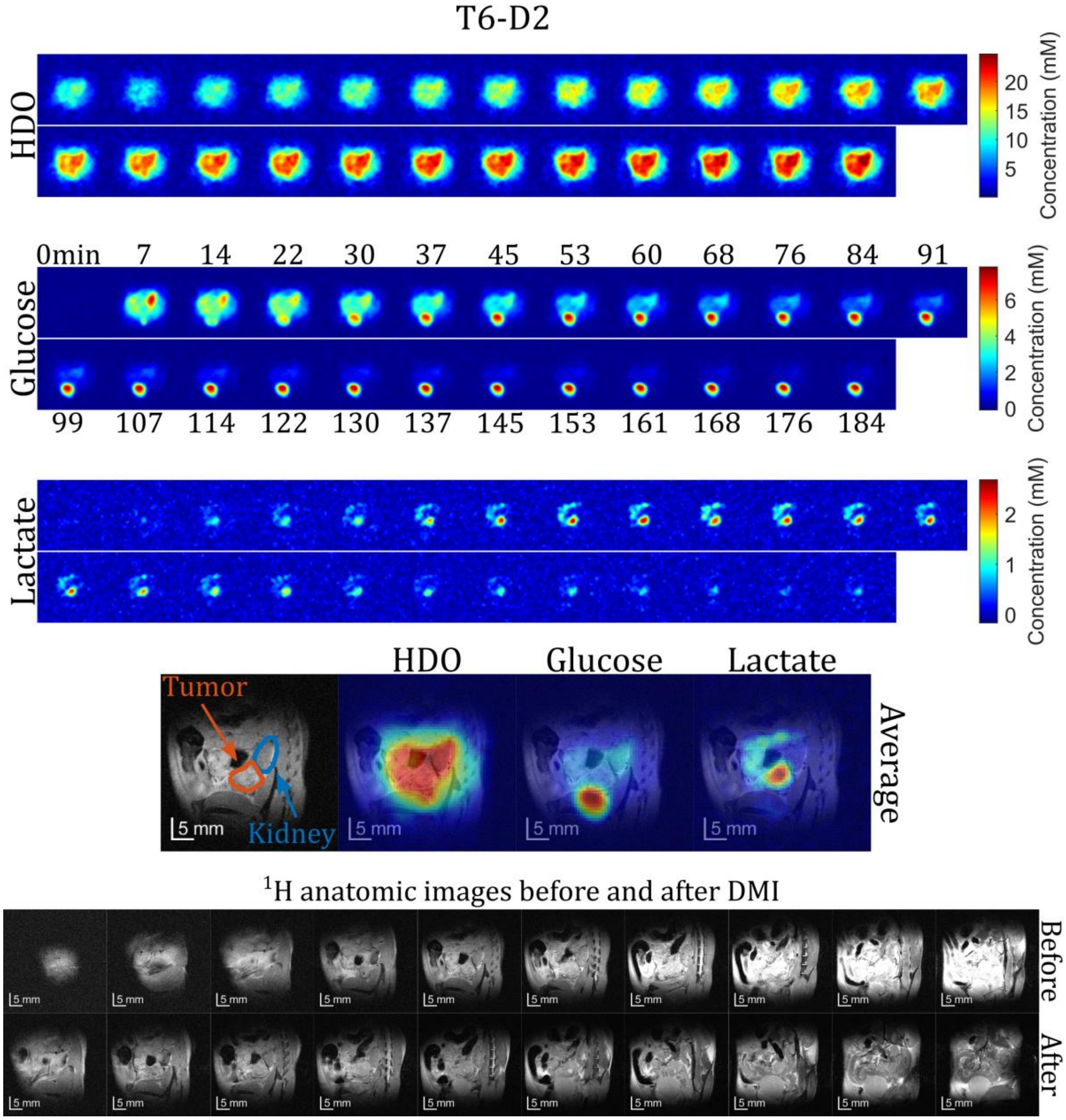
T6-D2
HDO
Concentration (mM)
20
15
10
5
0min
7
14
22
30
37
45
53
60
68
76
84
91
Glucose
Concentration (mM)
6
4
2
0
99
107
114
122
130
137
145
153
161
168
176
184
Lactate
Concentration (mM)
2
1
0
HDO
Glucose
Lactate
Tumor
Kidney
5 mm
Average
^{1}H anatomic images before and after DMI
Before
After

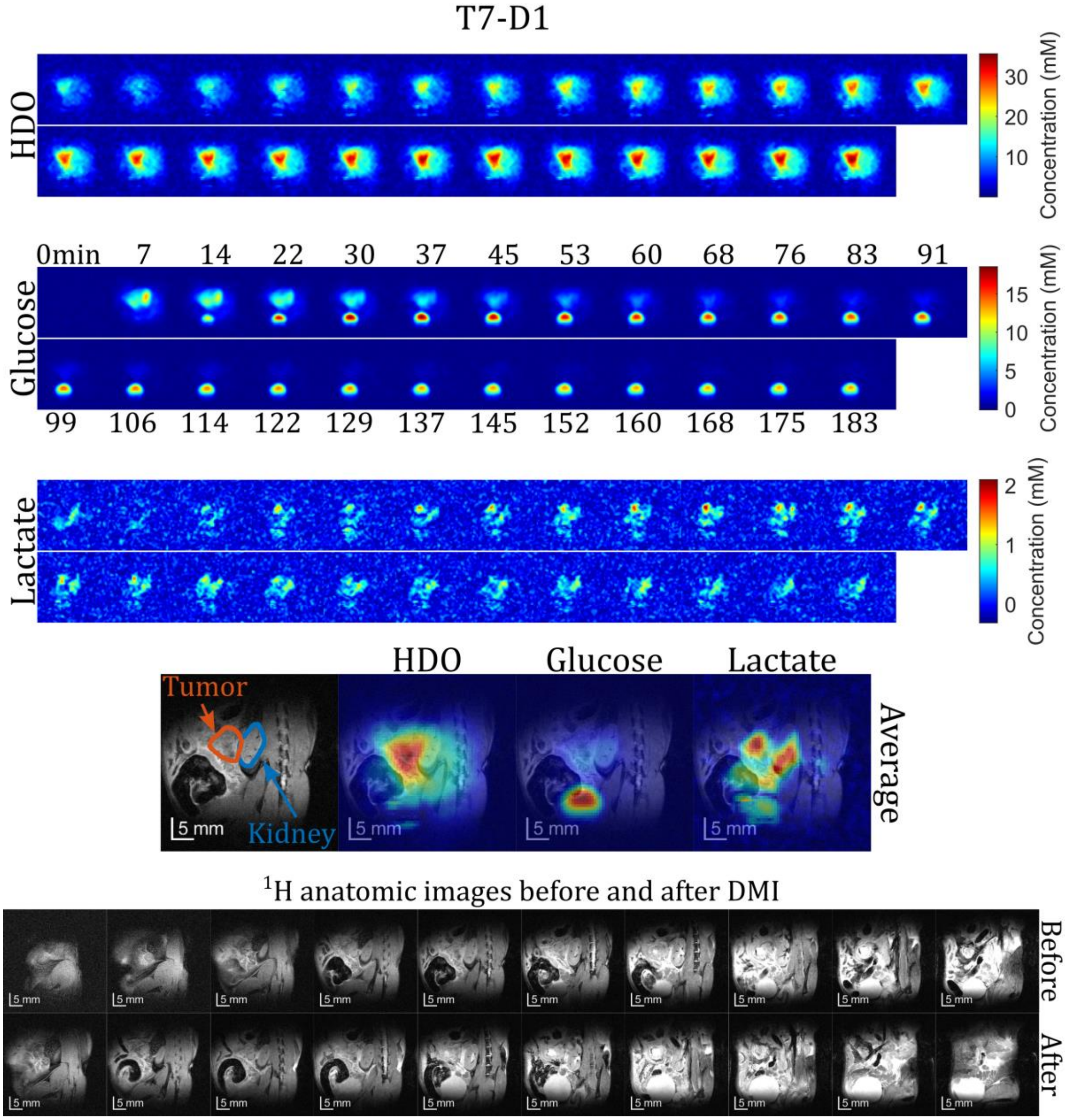

T7-D1
HDO
Glucose
Lactate
0min 7 14 22 30 37 45 53 60 68 76 83 91
99 106 114 122 129 137 145 152 160 168 175 183
Concentration (mM)
Tumor
Kidney
5 mm
Average
¹H anatomic images before and after DMI
Before
After

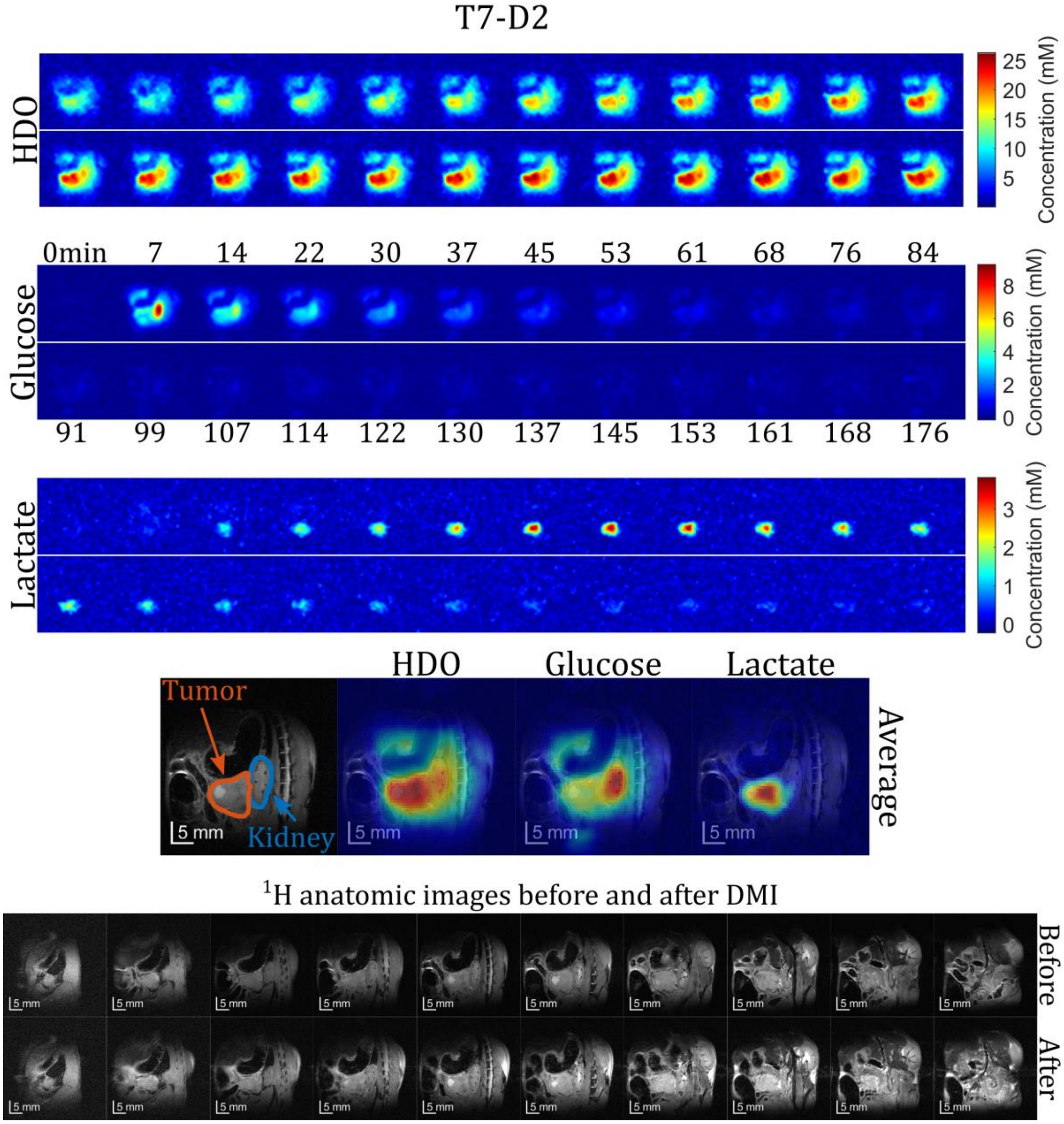
T7-D2
HDO
Glucose
Lactate
Concentration (mM)
25
20
15
10
5
8
6
4
2
0
3
2
1
0
0min
7
14
22
30
37
45
53
61
68
76
84
91
99
107
114
122
130
137
145
153
161
168
176
HDO
Glucose
Lactate
Tumor
Kidney
5 mm
Average
¹H anatomic images before and after DMI
Before
After

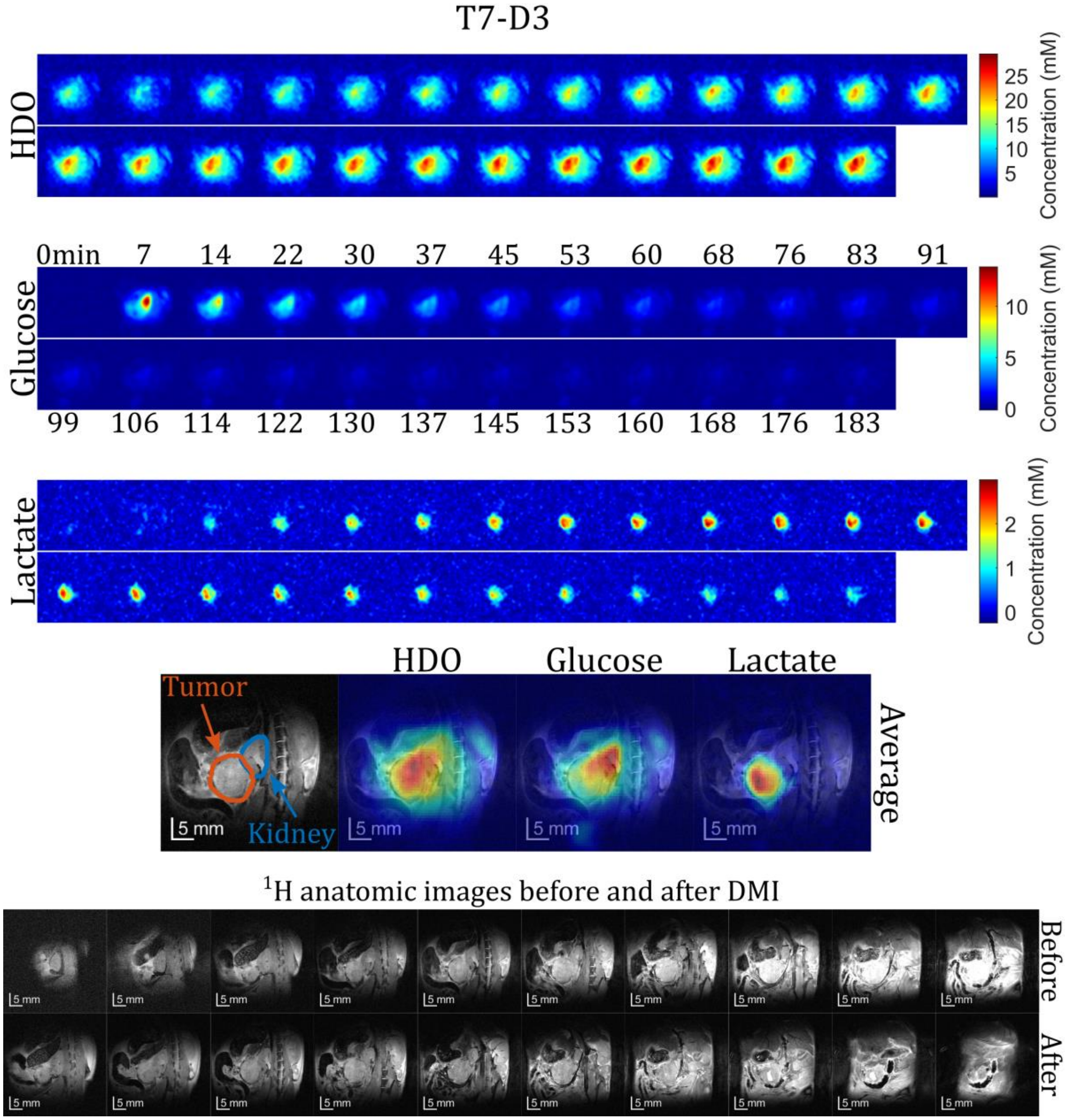

T7-D3
HDO
Glucose
Lactate
0min 7 14 22 30 37 45 53 60 68 76 83 91
99 106 114 122 130 137 145 153 160 168 176 183
Concentration (mM)
25 20 15 10 5
10 5 0
2 1 0
HDO Glucose Lactate
Average
Tumor
Kidney
5 mm
^{1}H anatomic images before and after DMI
Before
After

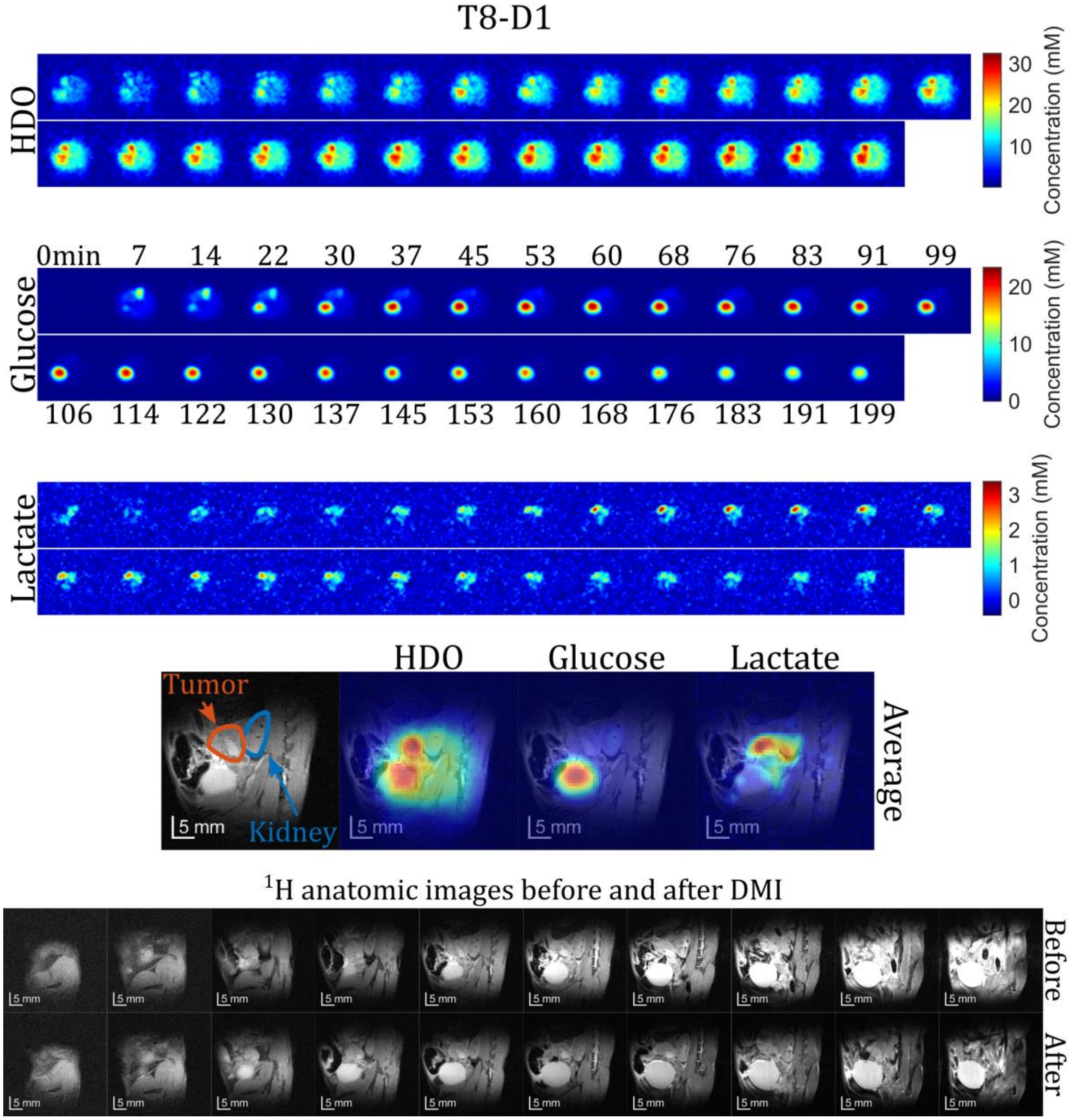
T8-D1
HDO
Concentration (mM)
30
20
10
0min
7
14
22
30
37
45
53
60
68
76
83
91
99
Glucose
20
10
0
106
114
122
130
137
145
153
160
168
176
183
191
199
Lactate
3
2
1
0
HDO
Glucose
Lactate
Tumor
Kidney
5 mm
Average
¹H anatomic images before and after DMI
Before
After

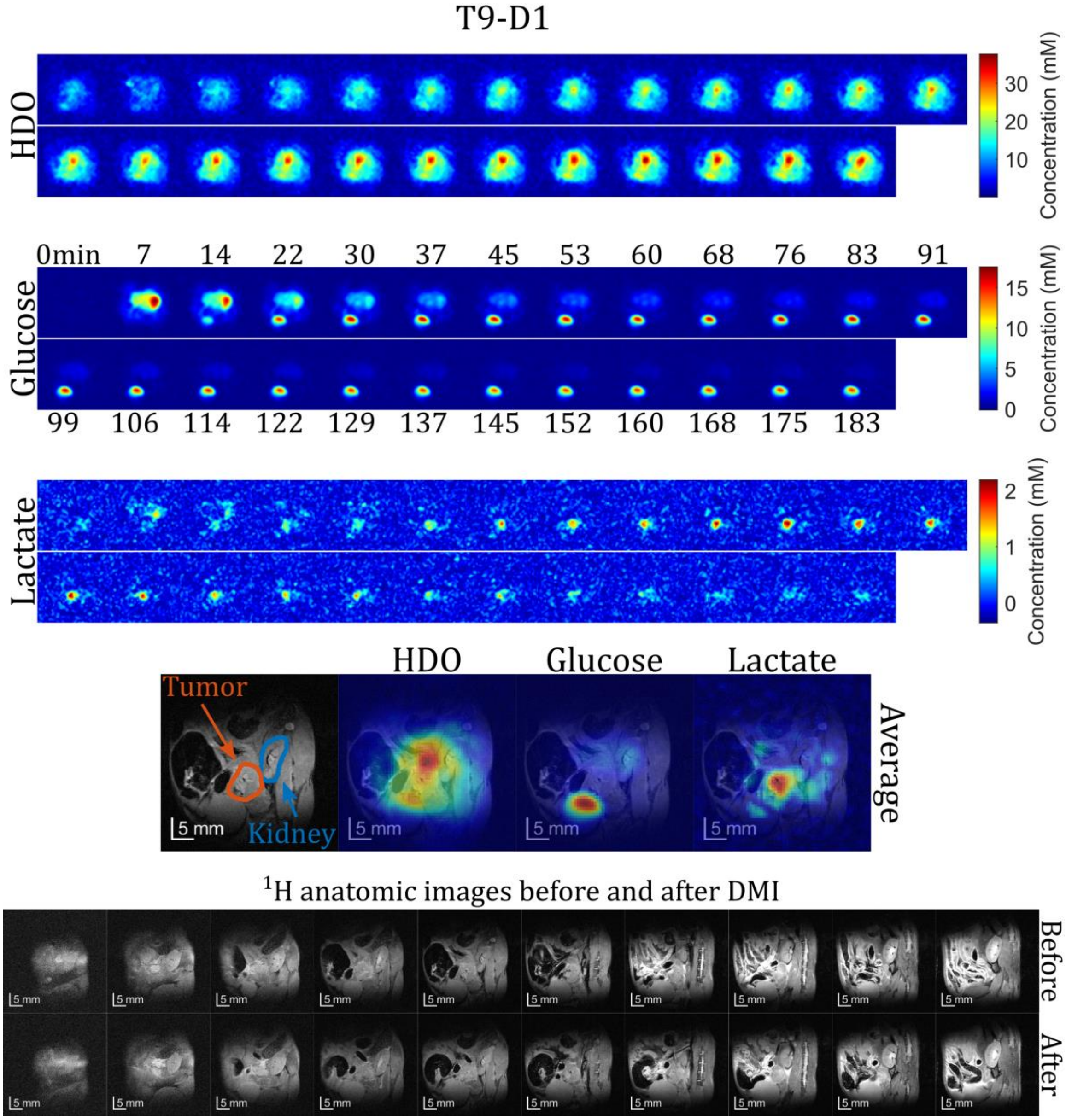
T9-D1
HDO
Concentration (mM)
0min
7
14
22
30
37
45
53
60
68
76
83
91
Glucose
99
106
114
122
129
137
145
152
160
168
175
183
Lactate
HDO
Glucose
Lactate
Tumor
Kidney
5 mm
Average
1H anatomic images before and after DMI
Before
After

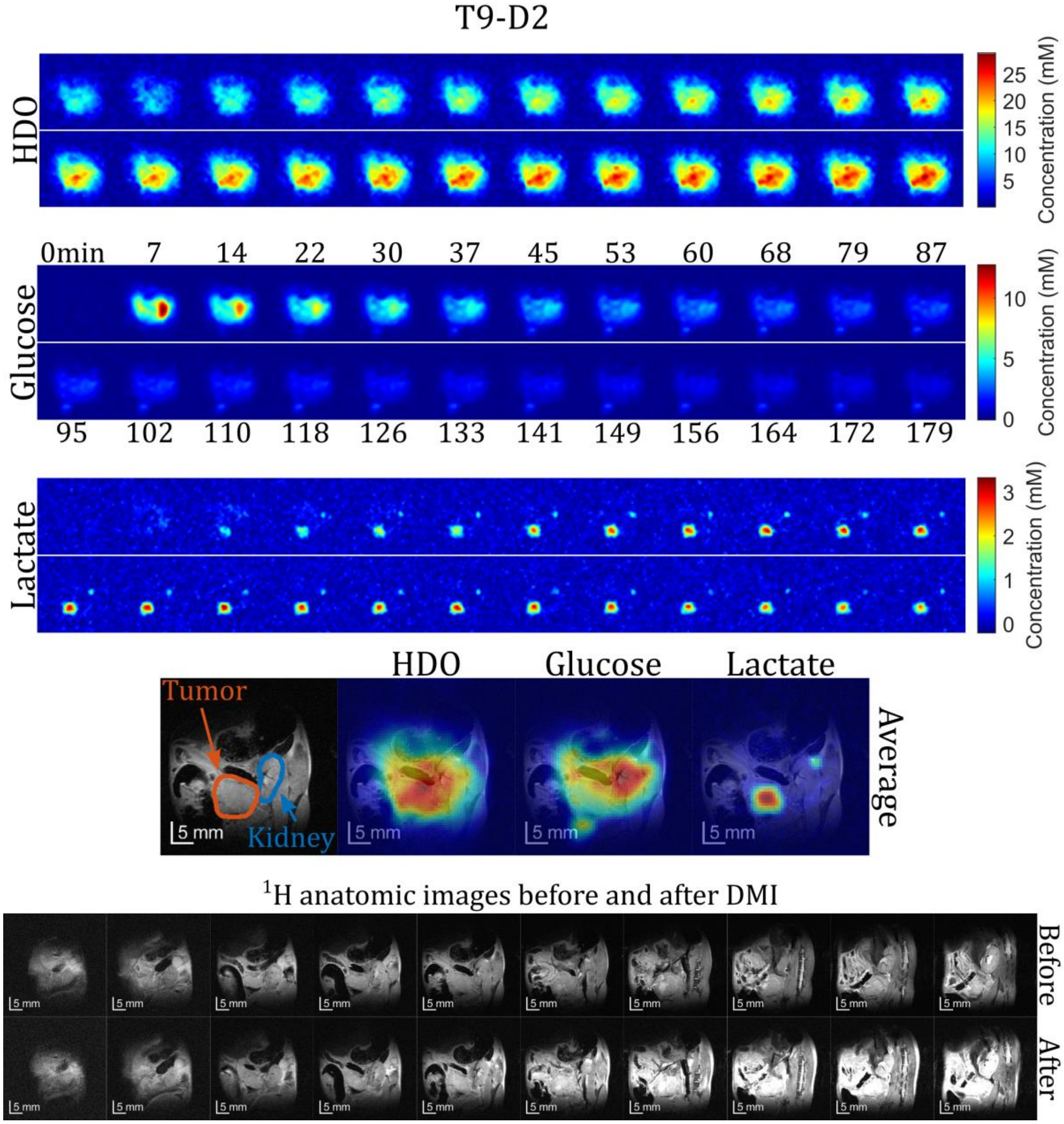
T9-D2
HDO
Concentration (mM)
25
20
15
10
5
0min
7
14
22
30
37
45
53
60
68
79
87
Glucose
Concentration (mM)
10
5
0
95
102
110
118
126
133
141
149
156
164
172
179
Lactate
Concentration (mM)
3
2
1
0
HDO
Glucose
Lactate
Tumor
Kidney
5 mm
Average
1H anatomic images before and after DMI
Before
After

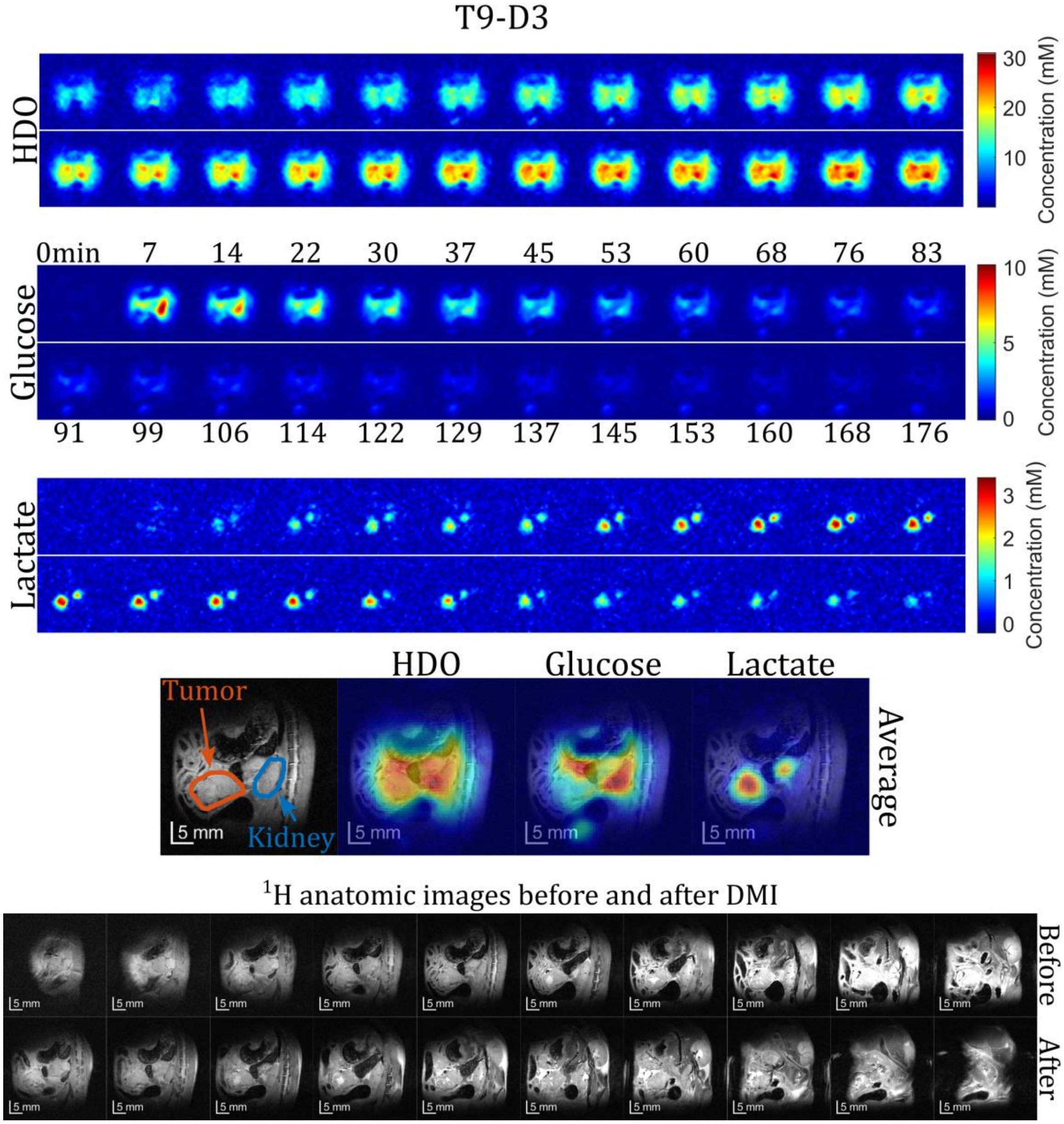
T9-D3
HDO
Glucose
Lactate
0min
7
14
22
30
37
45
53
60
68
76
83
91
99
106
114
122
129
137
145
153
160
168
176
Concentration (mM)
30
20
10
5
0
3
2
1
HDO
Glucose
Lactate
Tumor
Kidney
5 mm
Average
^{1}H anatomic images before and after DMI
Before
After

T1-D1

^{1}H anatomic images before and after DMI

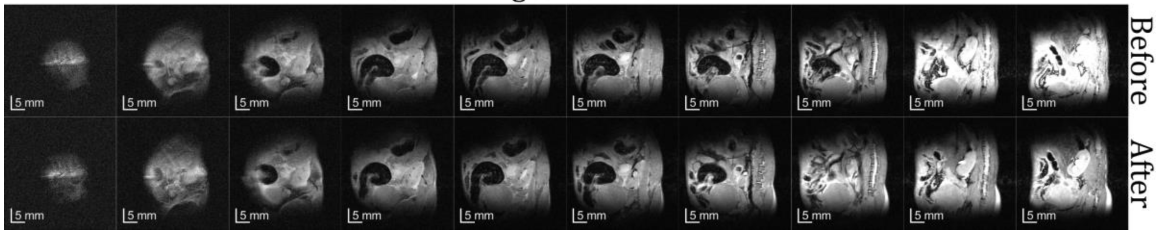

T2-D3

^{1}H anatomic images before and after DMI

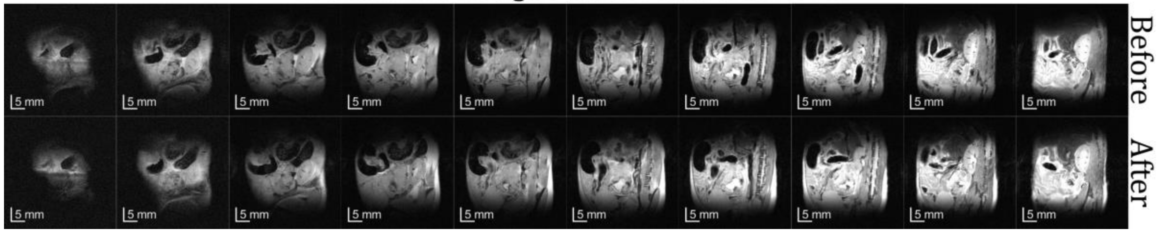

T4-D1

^{1}H anatomic images before and after DMI

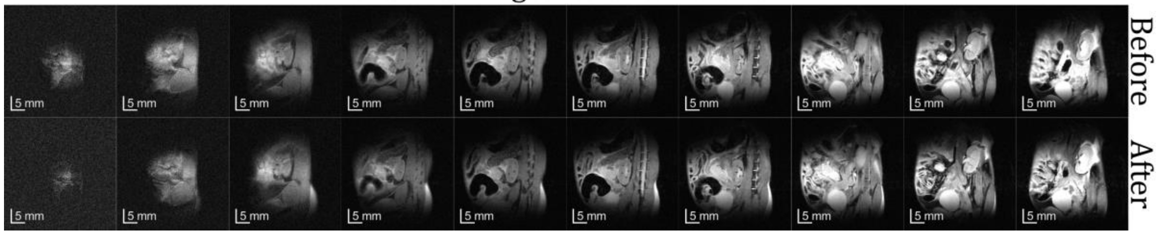

T4-D2

^{1}H anatomic images before and after DMI

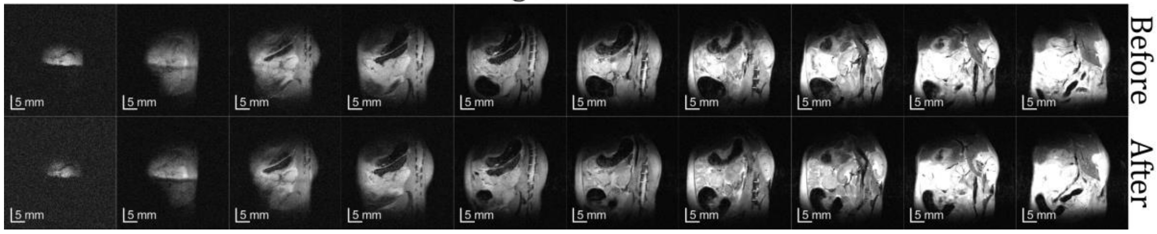

T5-D1

^{1}H anatomic images before and after DMI

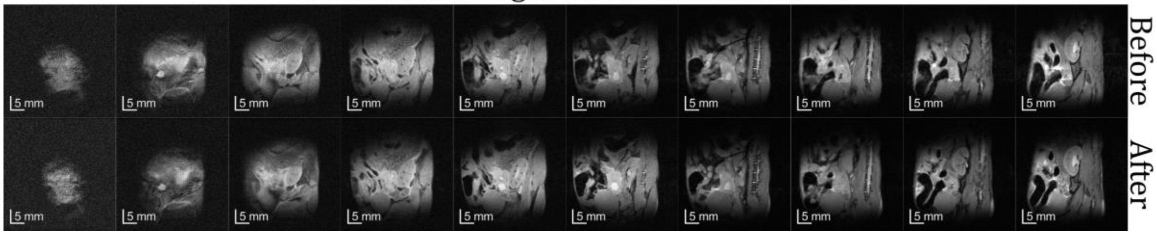

T5-D3

^{1}H anatomic images before and after DMI

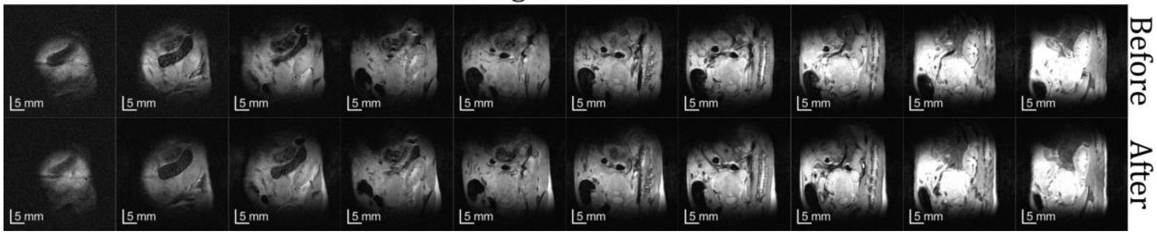

T8-D2

^{1}H anatomic images before and after DMI

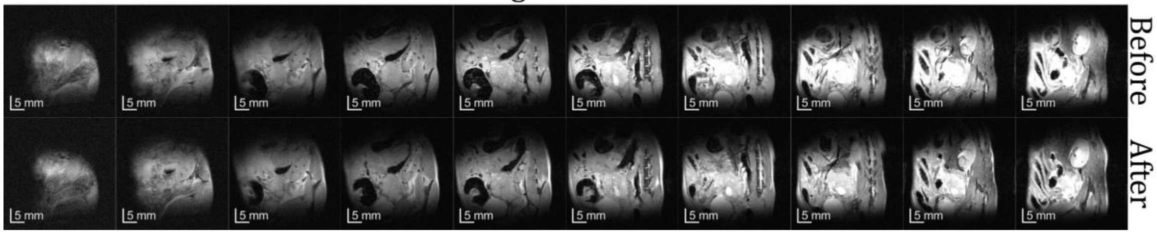

Section 2.2 Control Group

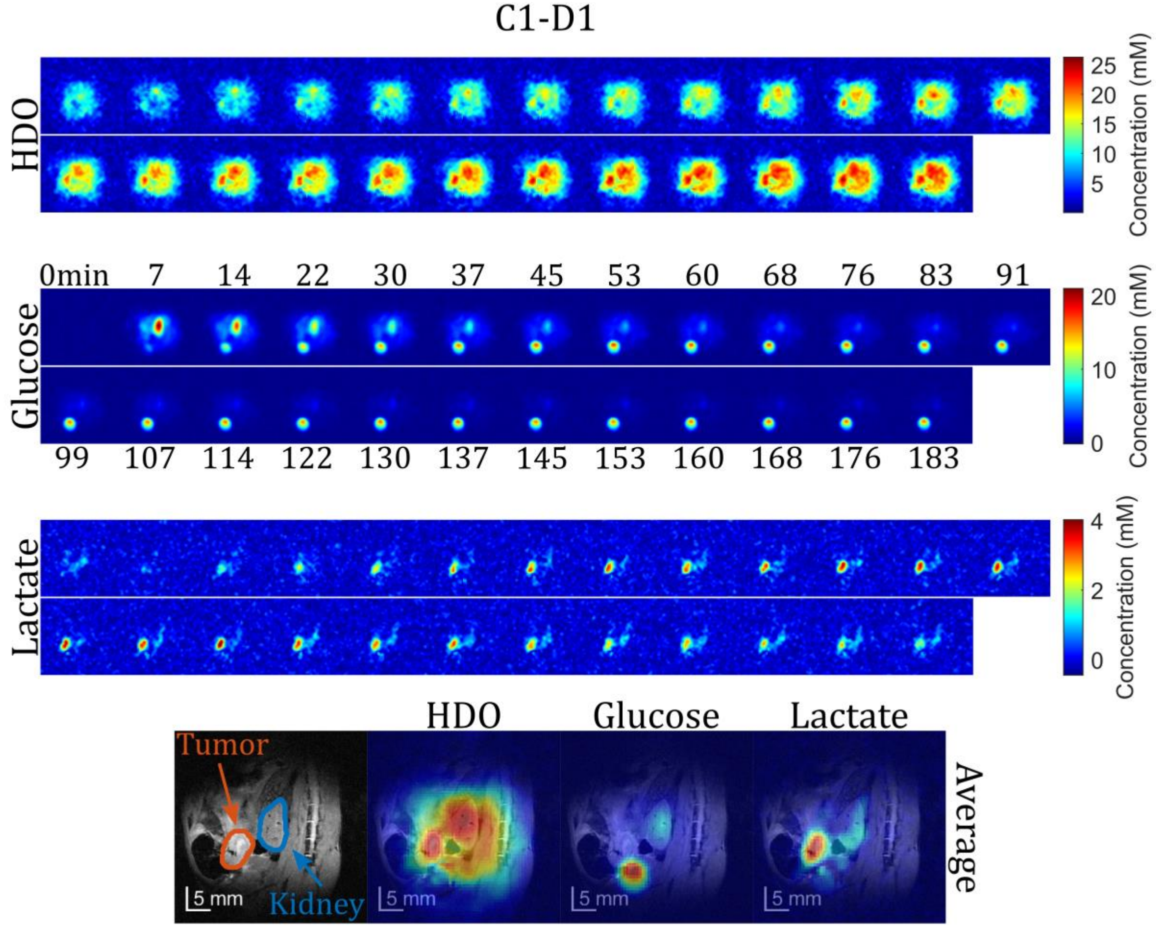

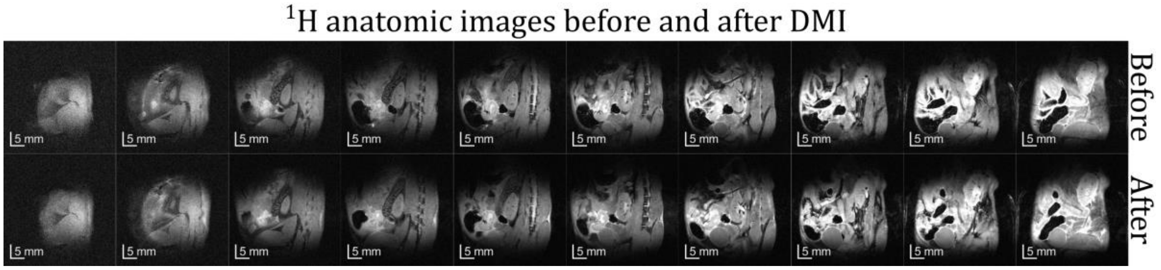

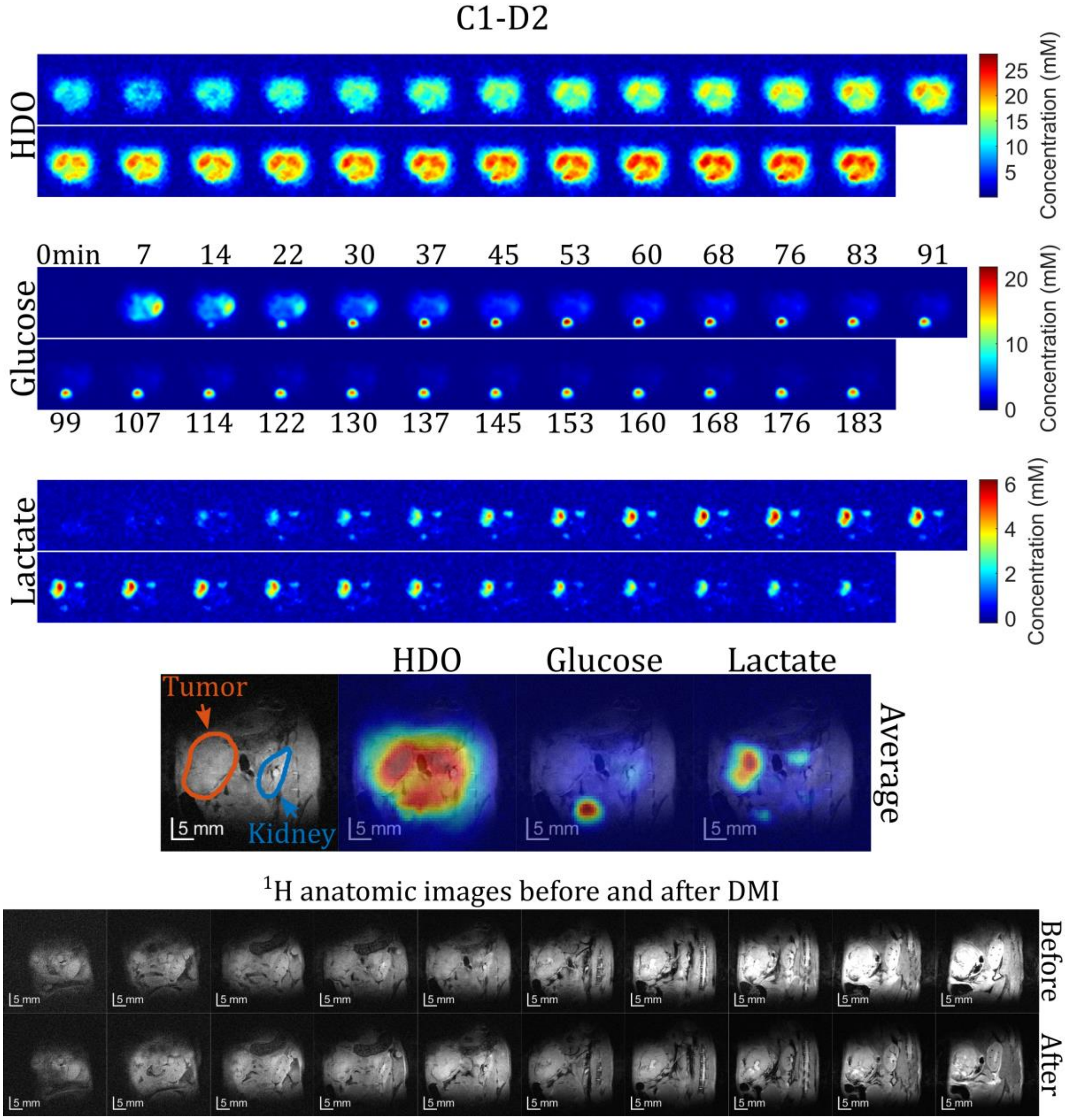
C1-D2
HDO
Concentration (mM)
25
20
15
10
5
0min
7
14
22
30
37
45
53
60
68
76
83
91
Glucose
20
10
0
99
107
114
122
130
137
145
153
160
168
176
183
Lactate
6
4
2
0
HDO
Glucose
Lactate
Tumor
Kidney
Average
5 mm
^{1}H anatomic images before and after DMI
Before
After

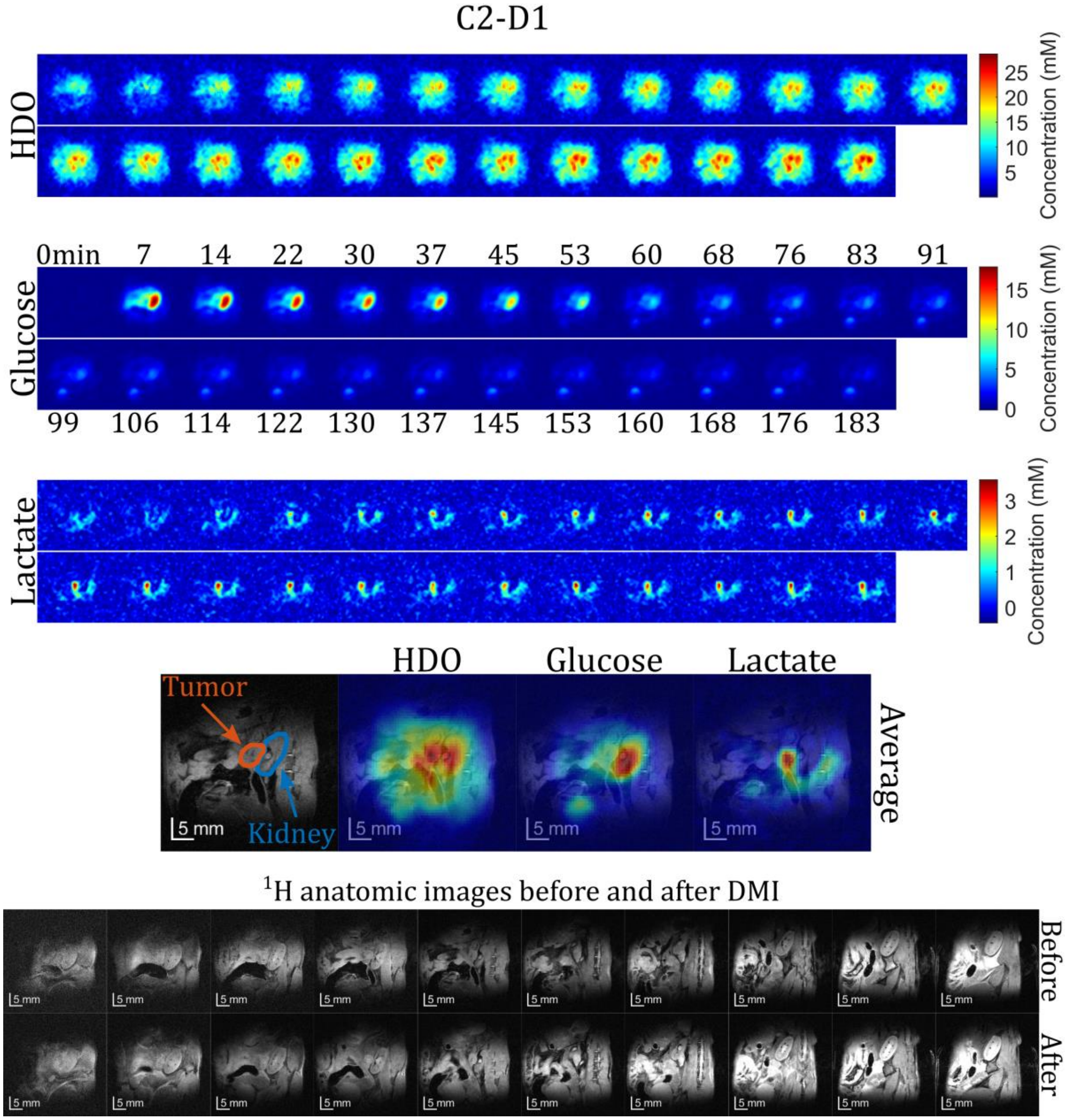
C2-D1
HDO
Glucose
Lactate
0min
7
14
22
30
37
45
53
60
68
76
83
91
99
106
114
122
130
137
145
153
160
168
176
183
Concentration (mM)
HDO
Glucose
Lactate
Tumor
Kidney
5 mm
Average
^{1}H anatomic images before and after DMI
Before
After

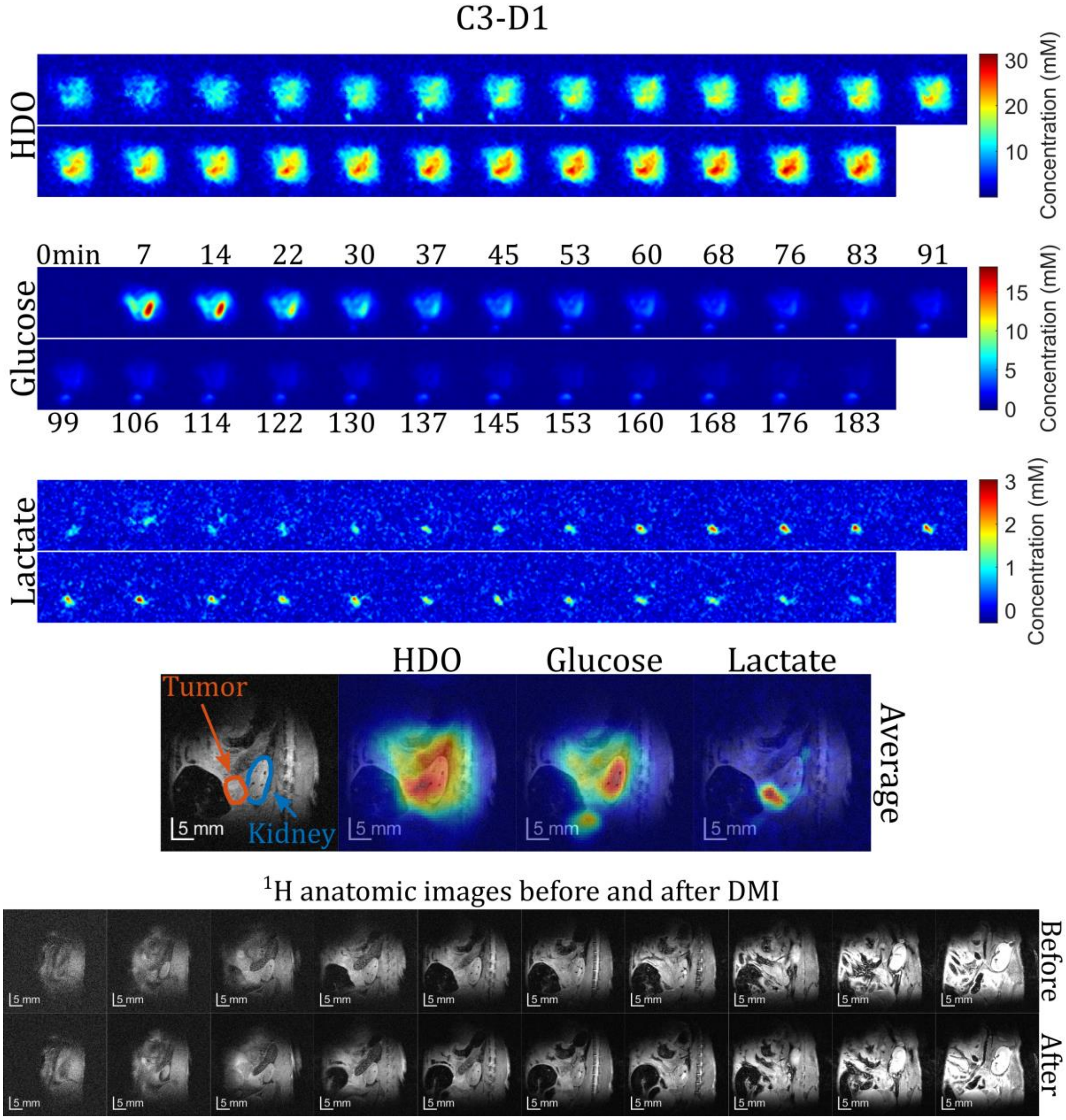
C3-D1
HDO
Concentration (mM)
30
20
10
0min
7
14
22
30
37
45
53
60
68
76
83
91
Glucose
15
10
5
0
99
106
114
122
130
137
145
153
160
168
176
183
Lactate
3
2
1
0
HDO
Glucose
Lactate
Tumor
Kidney
5 mm
Average
1H anatomic images before and after DMI
Before
After

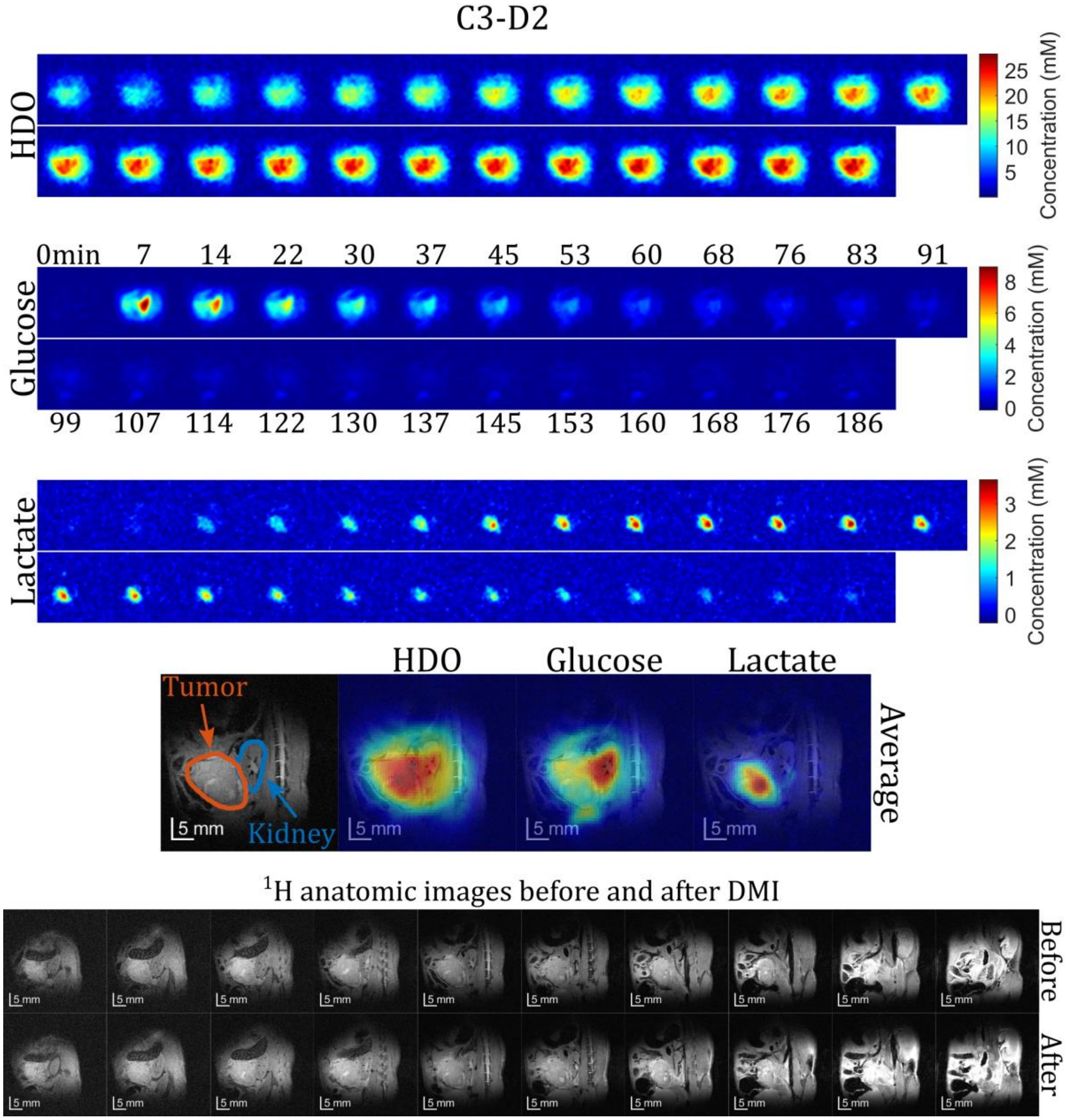
C3-D2
HDO
Concentration (mM)
25
20
15
10
5
0min
7
14
22
30
37
45
53
60
68
76
83
91
Glucose
8
6
4
2
0
99
107
114
122
130
137
145
153
160
168
176
186
Lactate
3
2
1
0
HDO
Glucose
Lactate
Tumor
Kidney
5 mm
Average
^{1}H anatomic images before and after DMI
Before
After

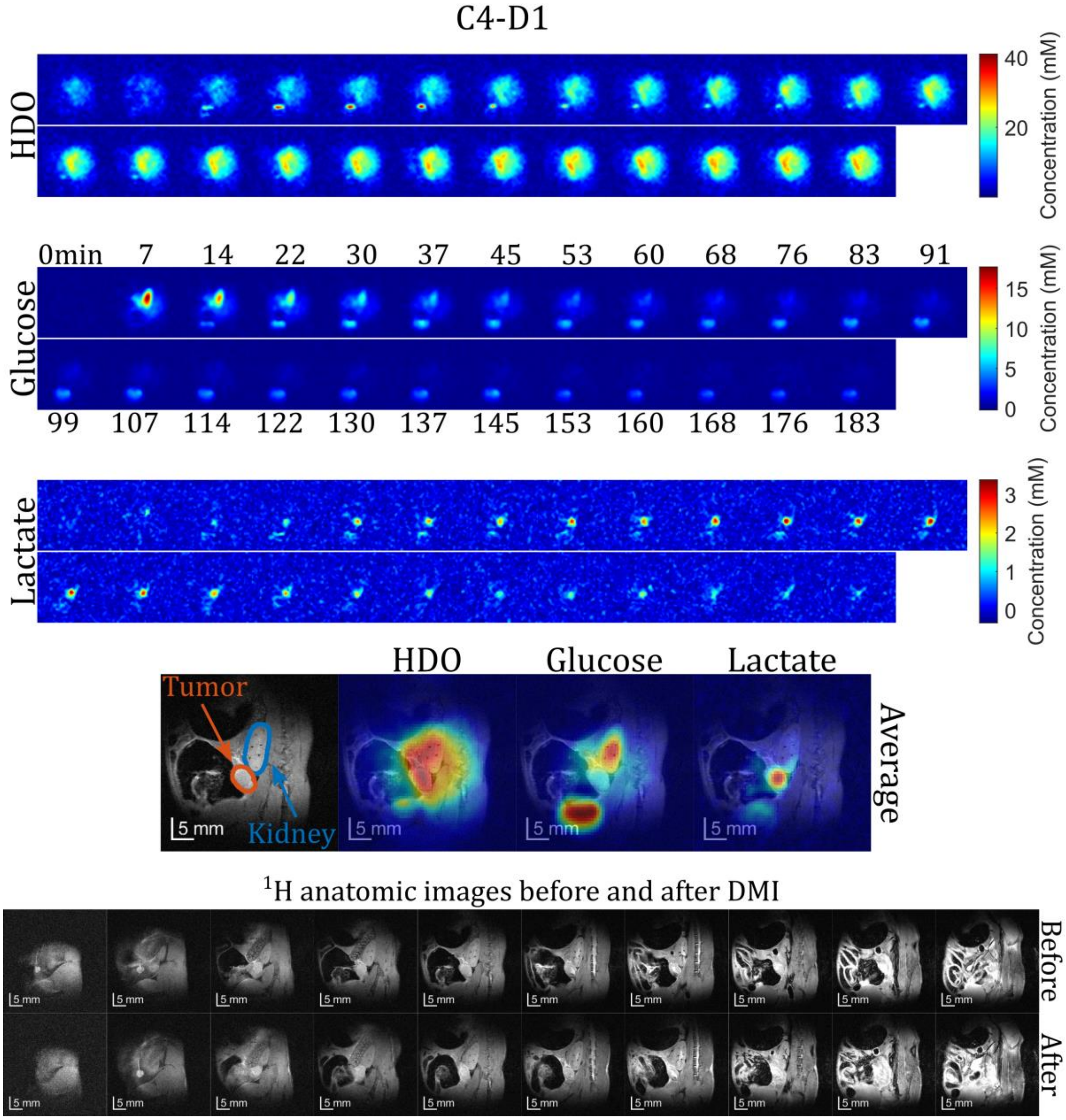

C4-D1
HDO
Glucose
Lactate
0min
7
14
22
30
37
45
53
60
68
76
83
91
99
107
114
122
130
137
145
153
160
168
176
183
Concentration (mM)
40
20
15
10
5
0
3
2
1
0
Tumor
Kidney
5 mm
HDO
Glucose
Lactate
Average
1H anatomic images before and after DMI
Before
After

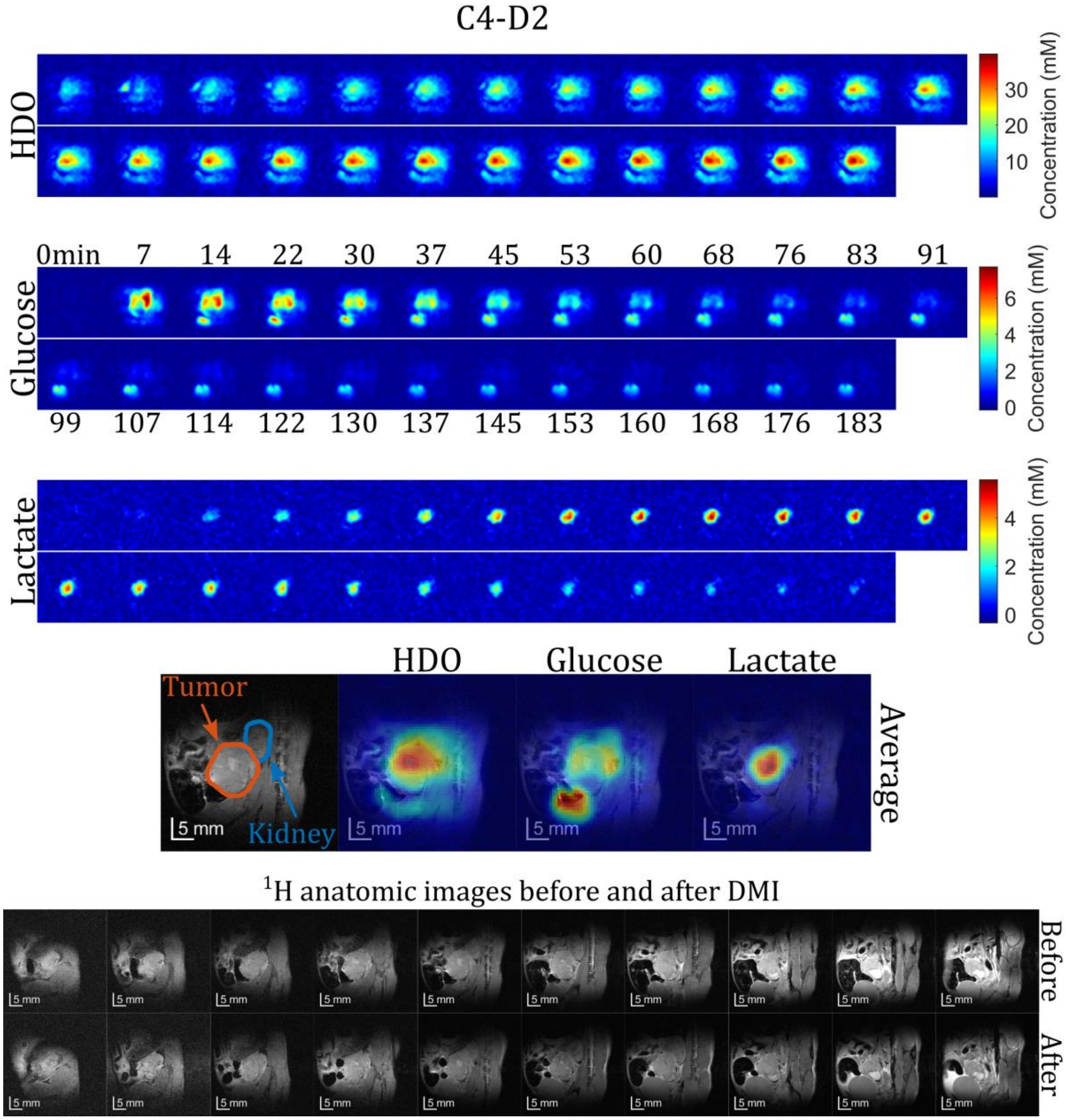

C4-D2
HDO
Glucose
Lactate
Concentration (mM)
0min
7
14
22
30
37
45
53
60
68
76
83
91
99
107
114
122
130
137
145
153
160
168
176
183
HDO
Glucose
Lactate
Tumor
Kidney
5 mm
Average
^{1}H anatomic images before and after DMI
Before
After

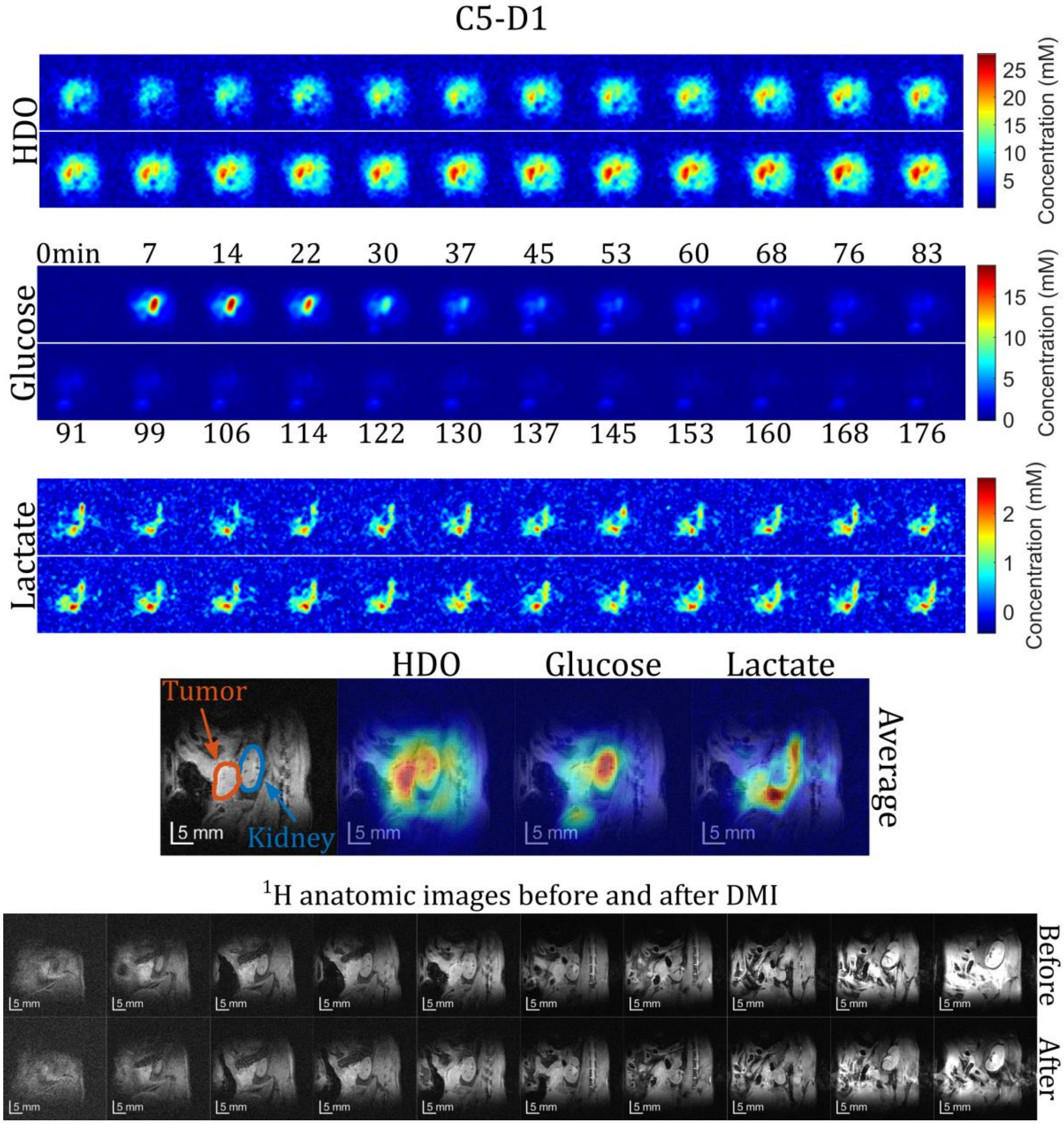
C5-D1
HDO
Glucose
Lactate
Concentration (mM)
0min 7 14 22 30 37 45 53 60 68 76 83
91 99 106 114 122 130 137 145 153 160 168 176
HDO Glucose Lactate
Tumor
Kidney
5 mm
Average
1H anatomic images before and after DMI
Before
After

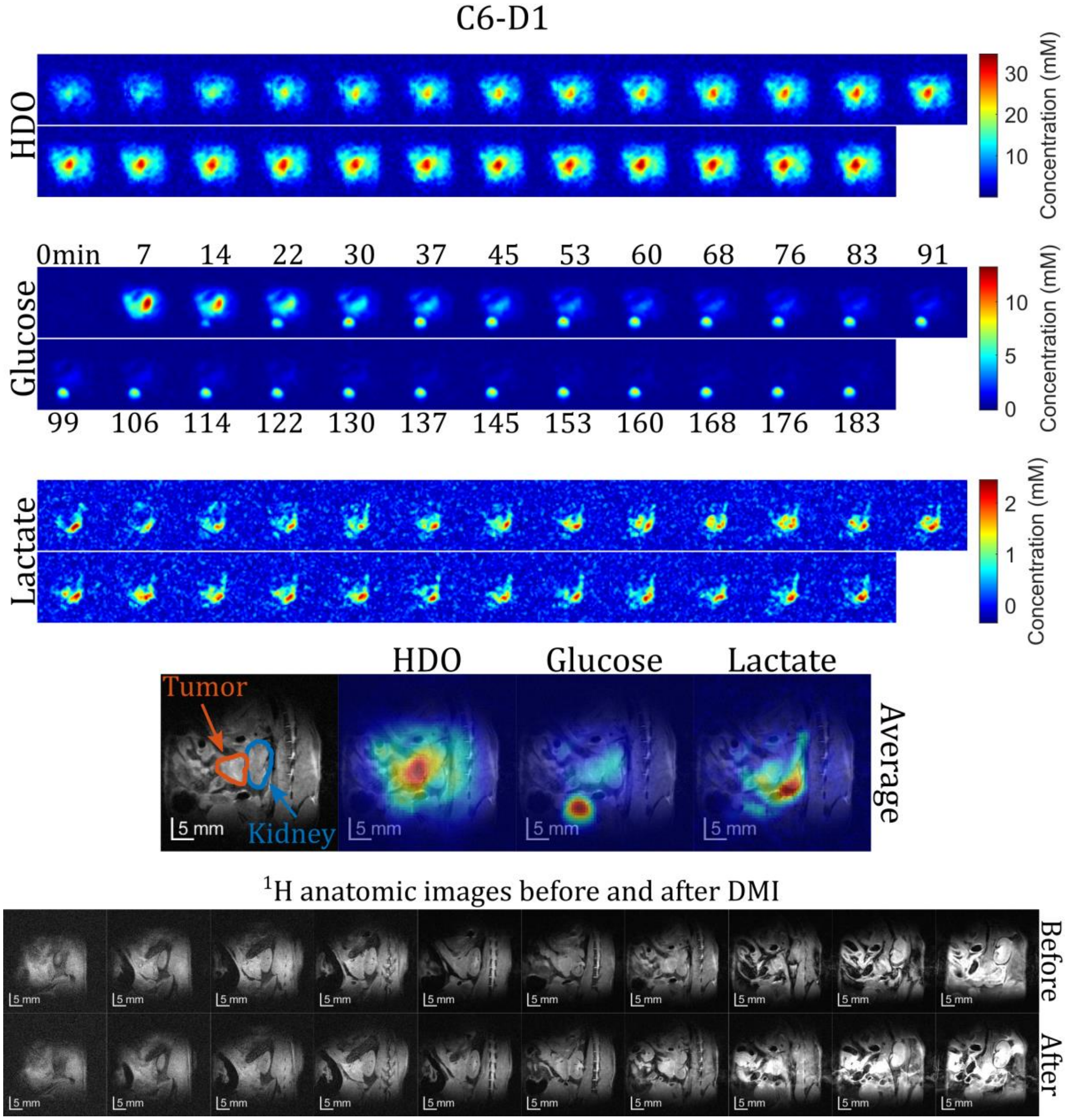
C6-D1
HDO
Glucose
Lactate
0min 7 14 22 30 37 45 53 60 68 76 83 91
99 106 114 122 130 137 145 153 160 168 176 183
Concentration (mM)
Tumor
Kidney
5 mm
HDO Glucose Lactate
Average
¹H anatomic images before and after DMI
Before
After

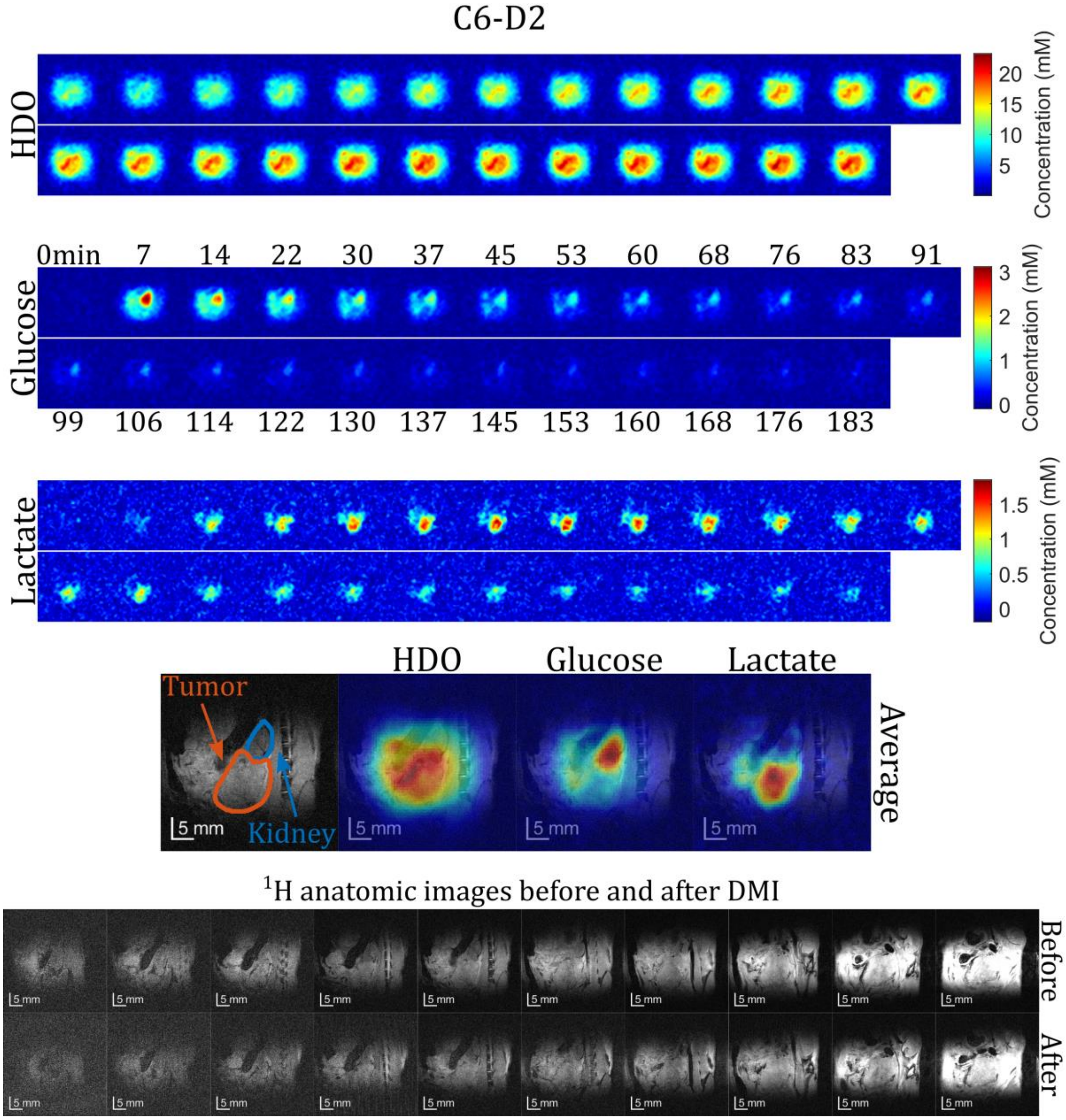
C6-D2
HDO
Glucose
Lactate
0min
7
14
22
30
37
45
53
60
68
76
83
91
99
106
114
122
130
137
145
153
160
168
176
183
Concentration (mM)
20
15
10
5
Concentration (mM)
3
2
1
0
Concentration (mM)
1.5
1
0.5
0
HDO
Glucose
Lactate
Tumor
Kidney
5 mm
Average
^{1}H anatomic images before and after DMI
Before
After

Section 3: Summary of all experimental ^2H MRSI (lactate, glucose) data fits for every animal and scan.

The panels in the following eight-pages Figure summarizes the kinetic fits for the Treated and Control datasets collected by DMI, taking volume-normalized concentrations of healthy (kidney) and tumor ROIs. These data lead to the statistics shown in Figures 5 and 6 of the main text, and the kinetic parameters are summarized in Section 3.

Section 3.1 Treatment Group

T1-D2

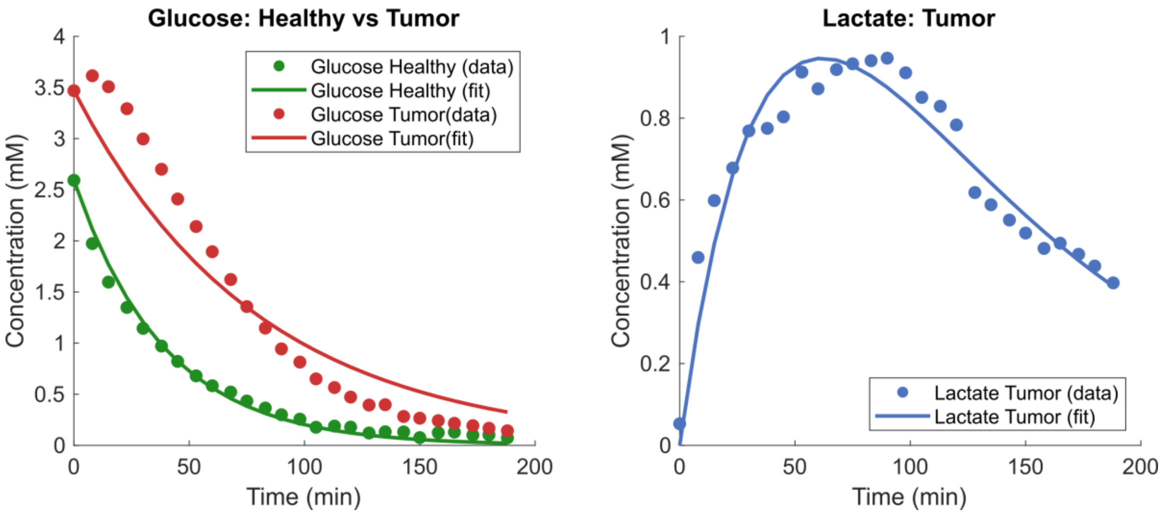

T2-D1

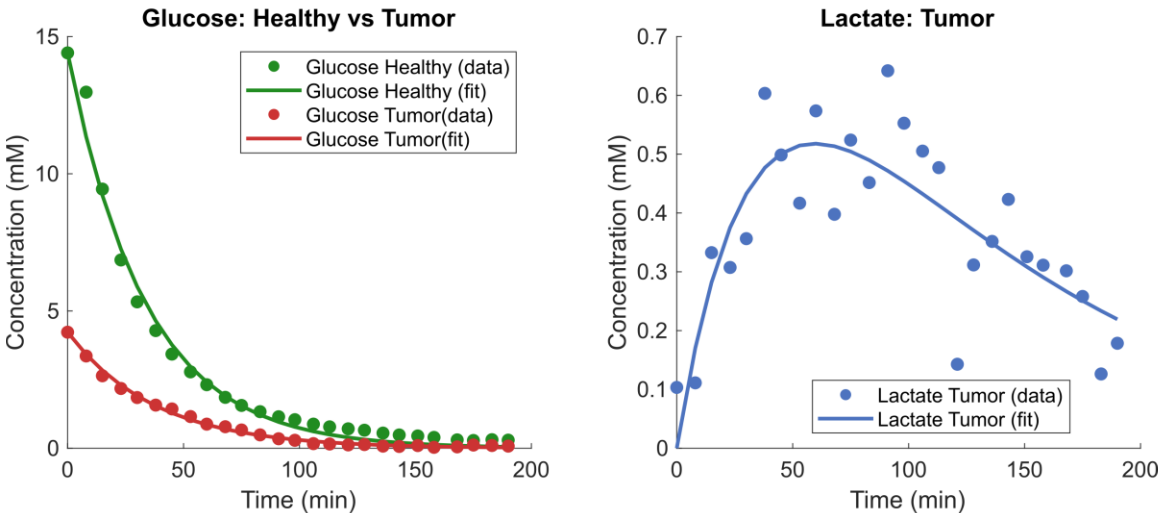

T2-D2

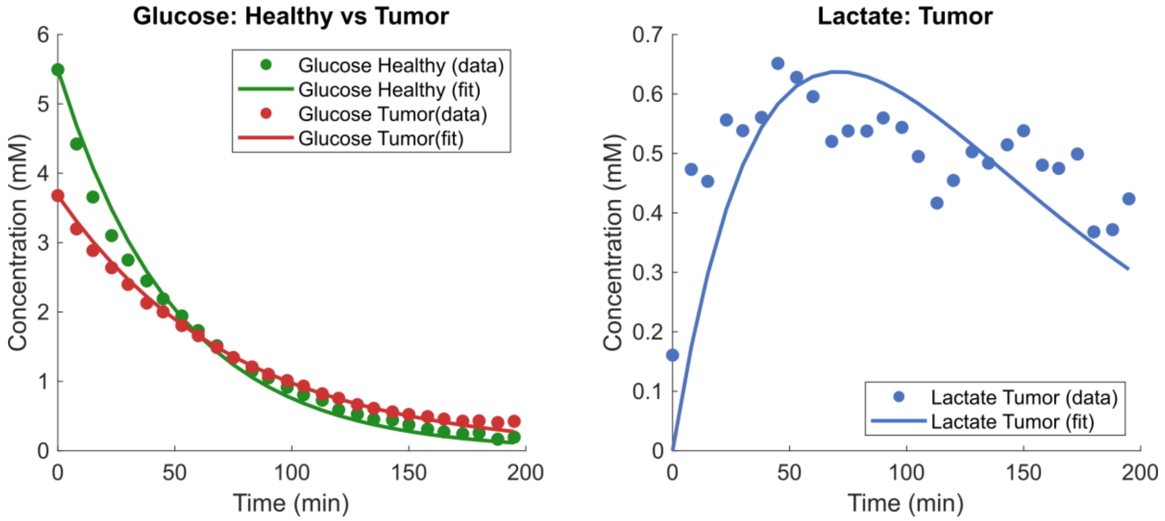

T3-D1

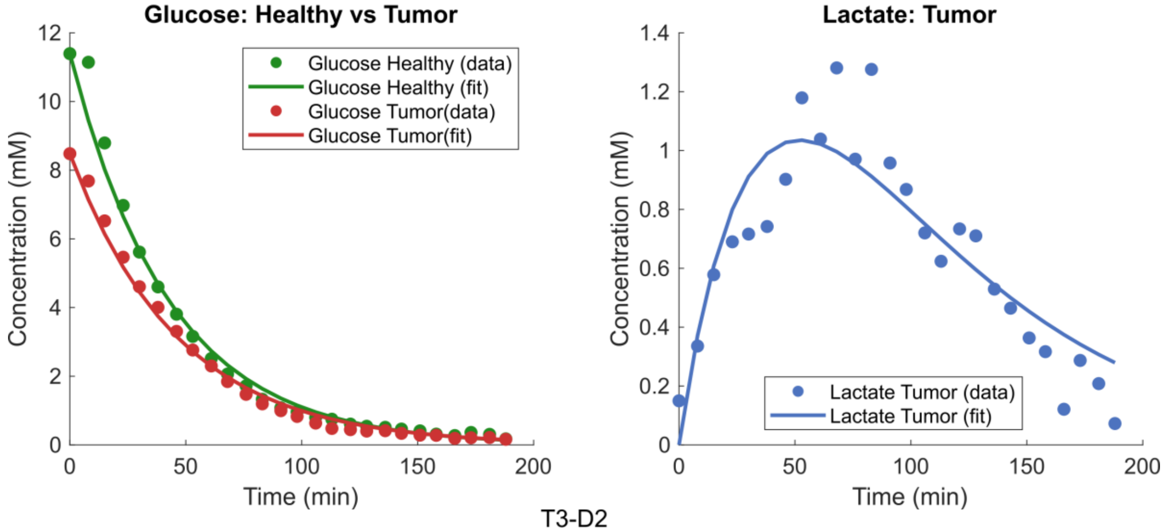

T3-D2

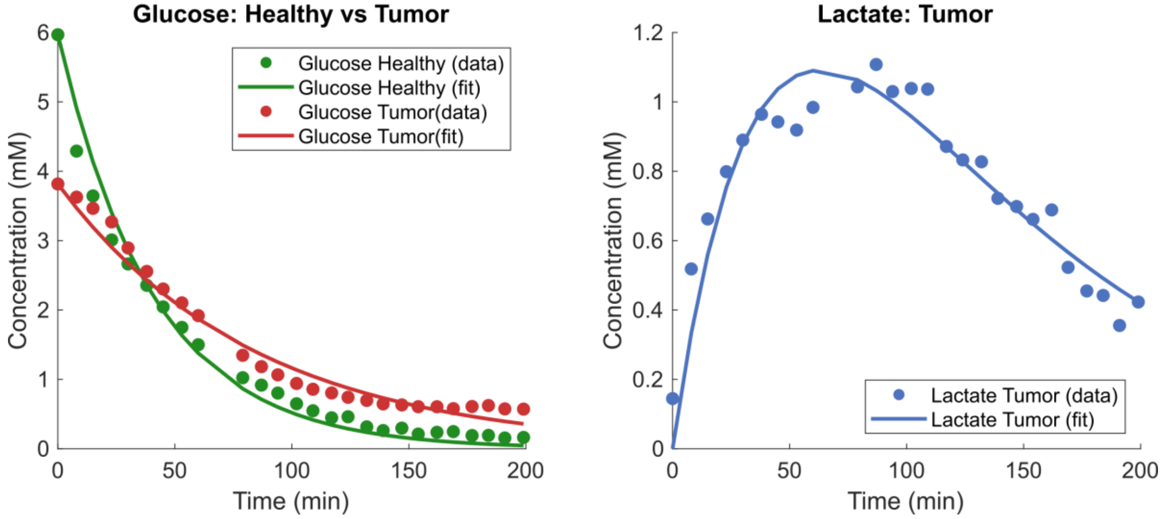

T5-D2

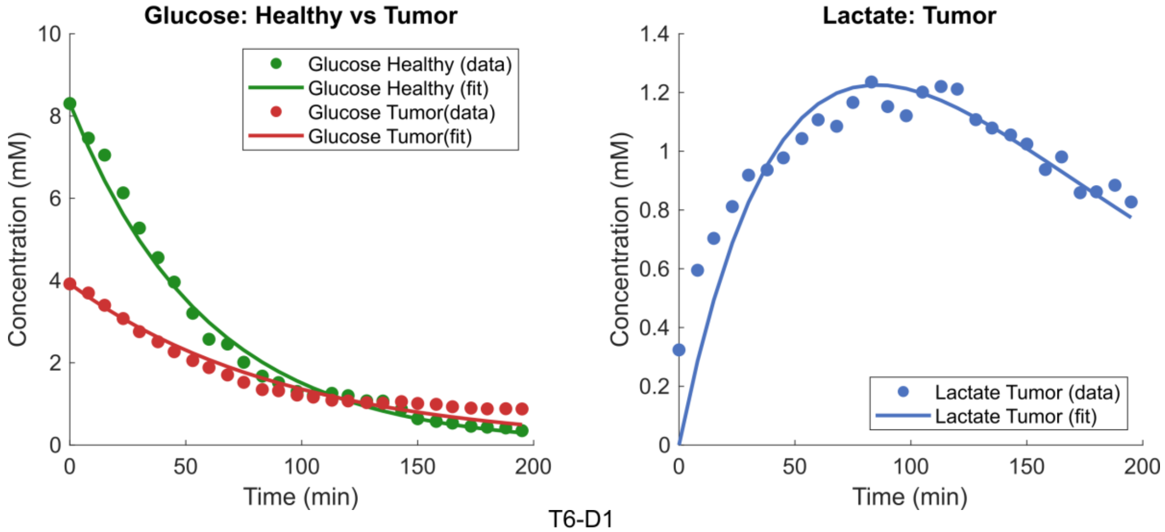

T6-D1

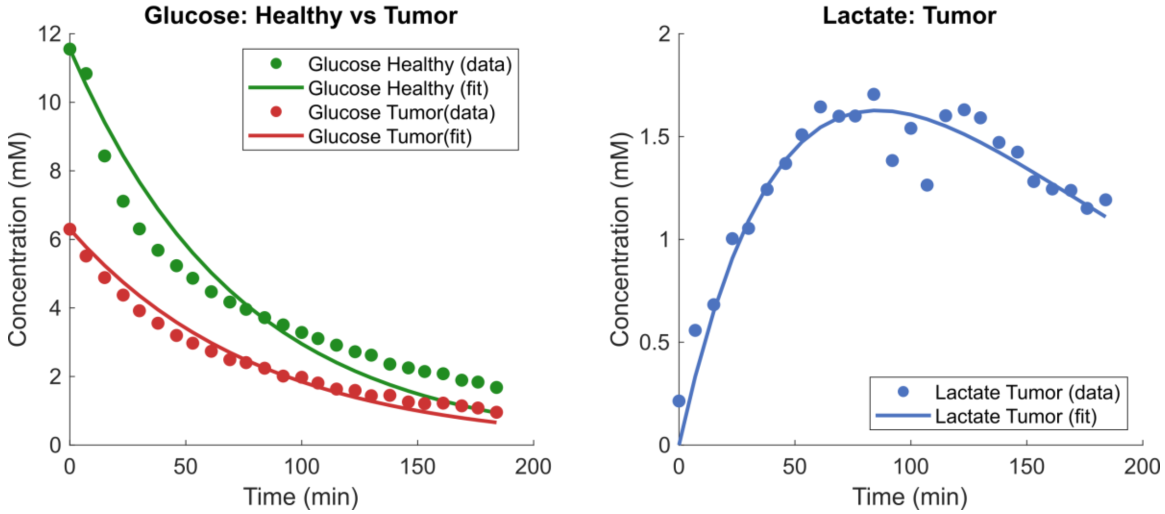

T6-D2

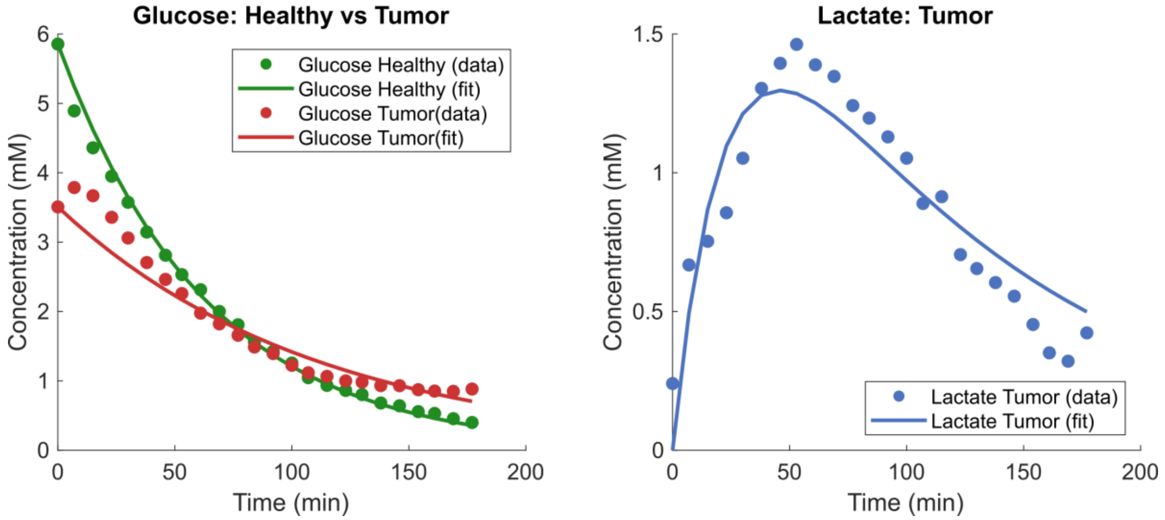

T7-D1

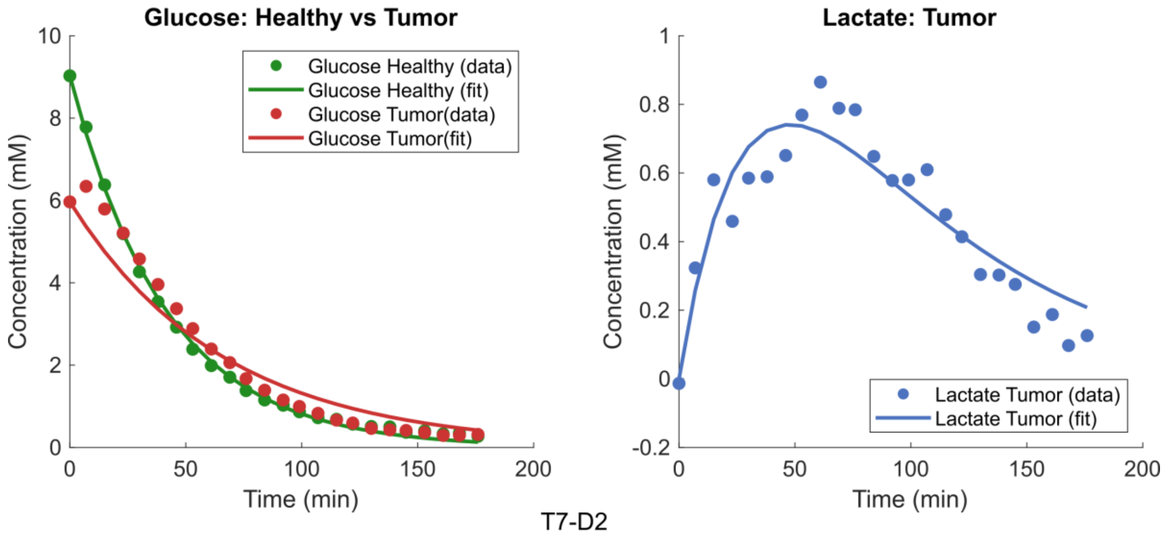

T7-D2

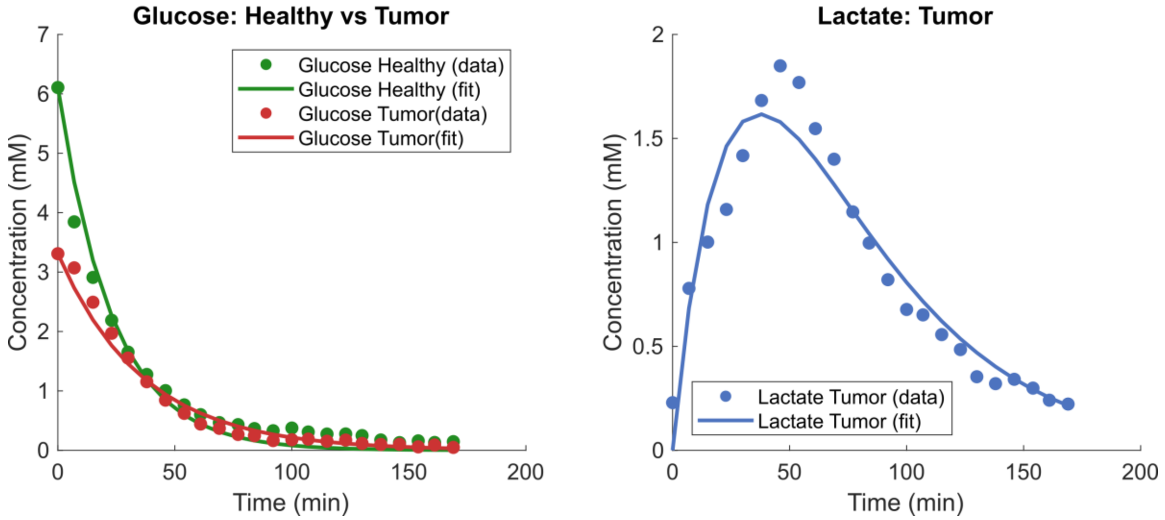

T7-D3

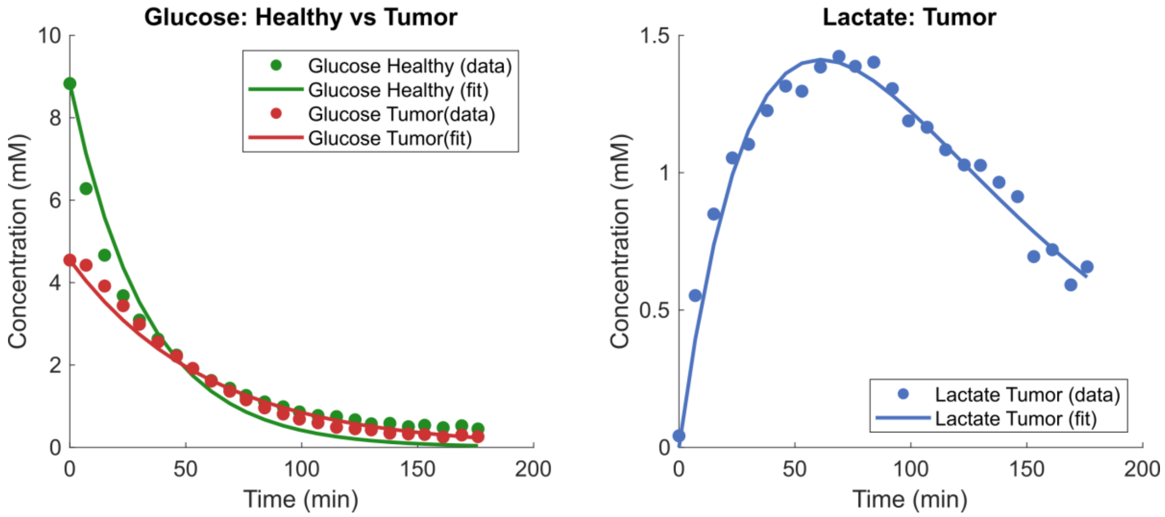

T8-D1

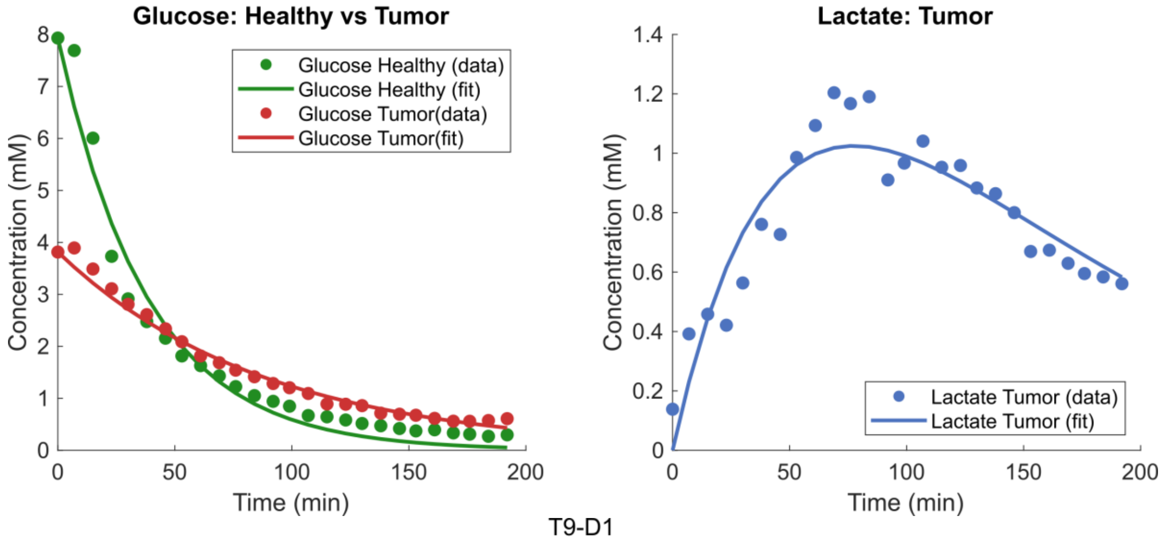

T9-D1

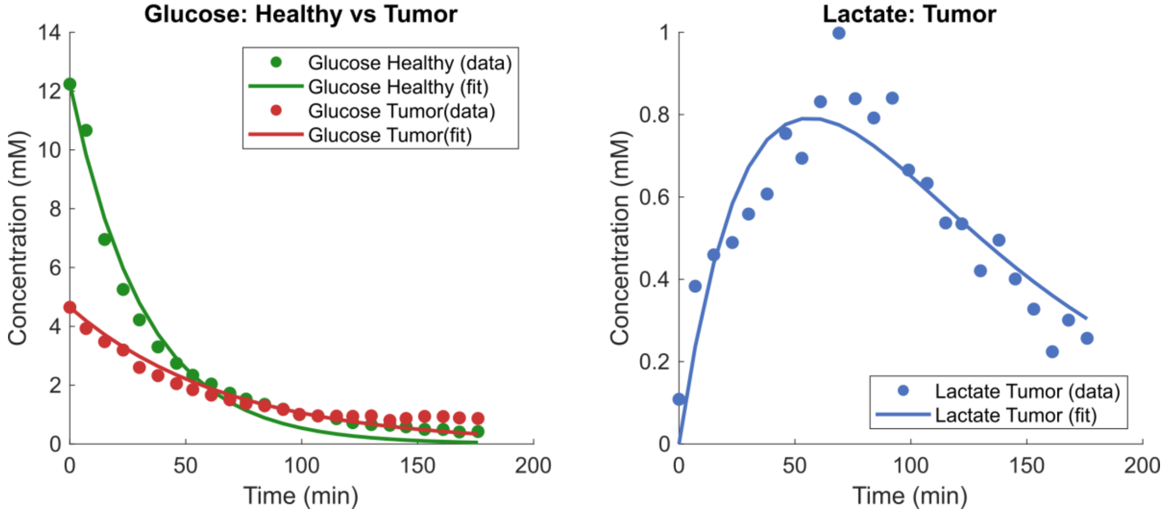

T9-D2

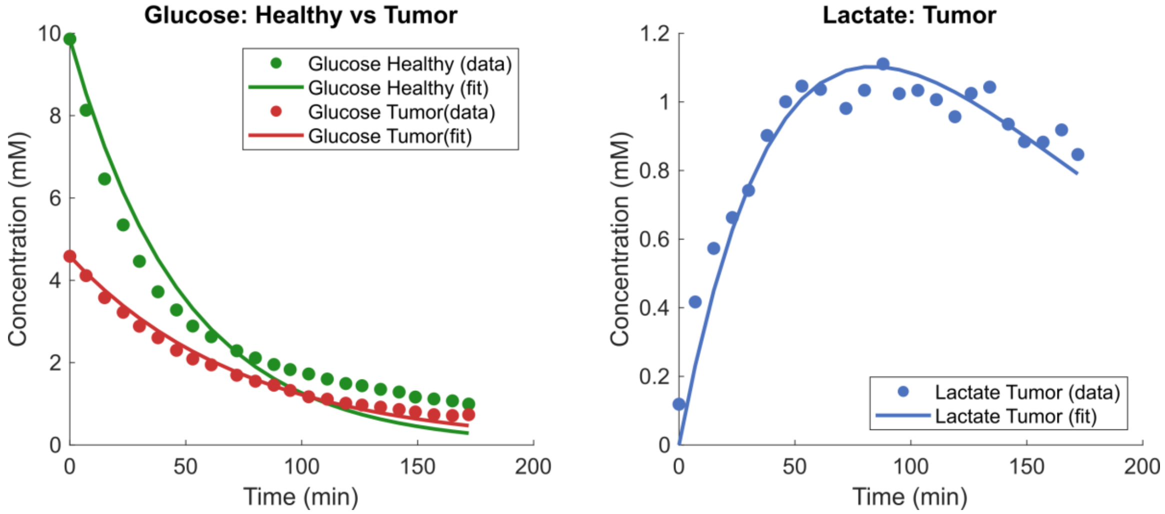

T9-D3

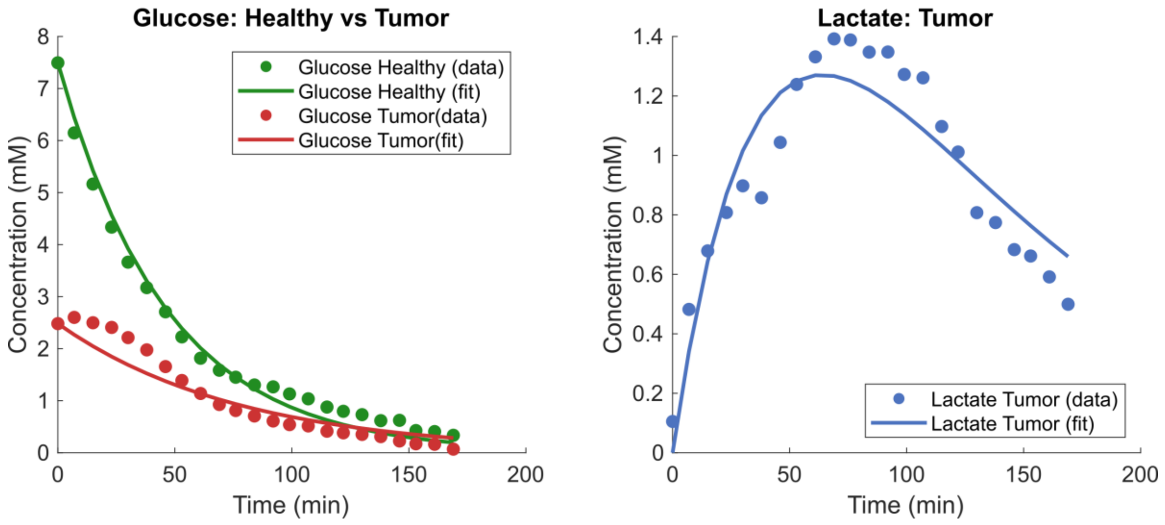

Section 3.2 Control Group

C1-D1

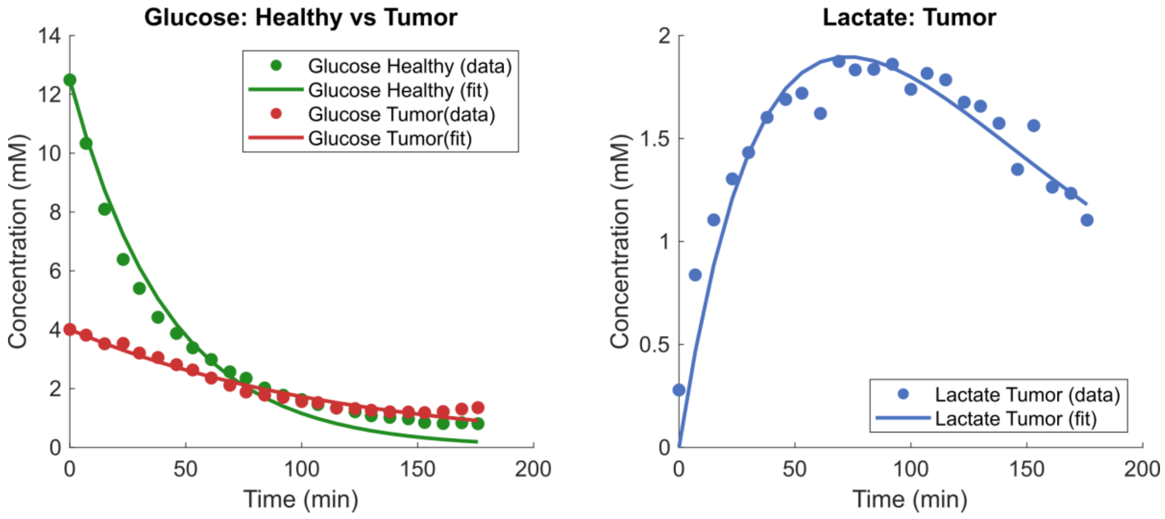

C1-D2

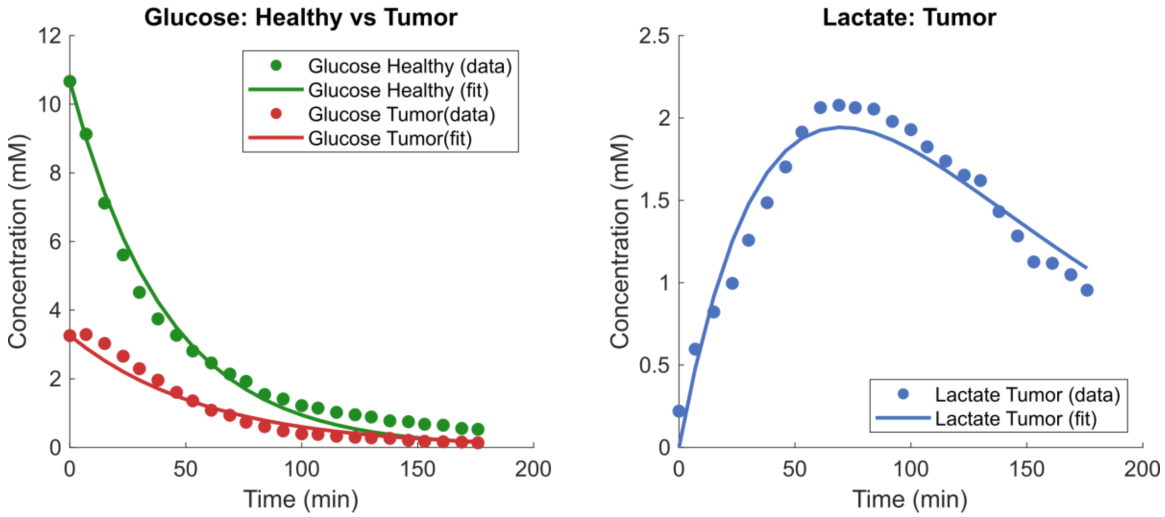

C2-D1

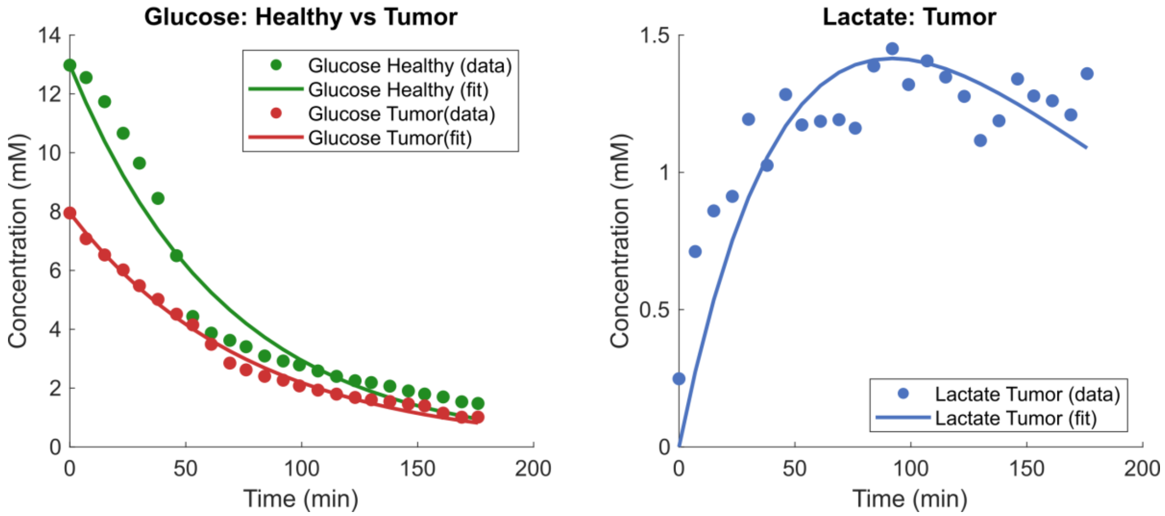

C3-D1

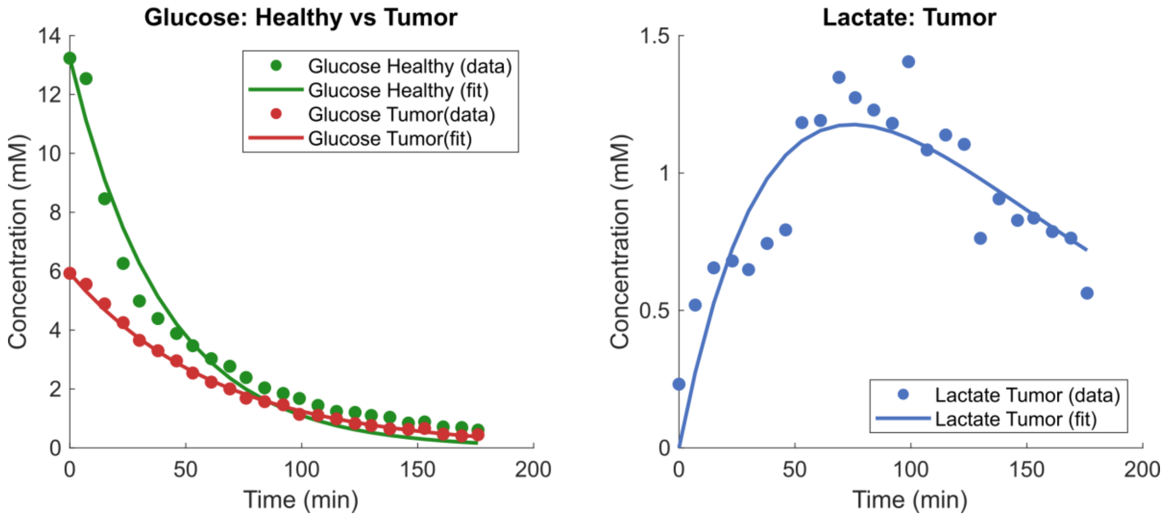

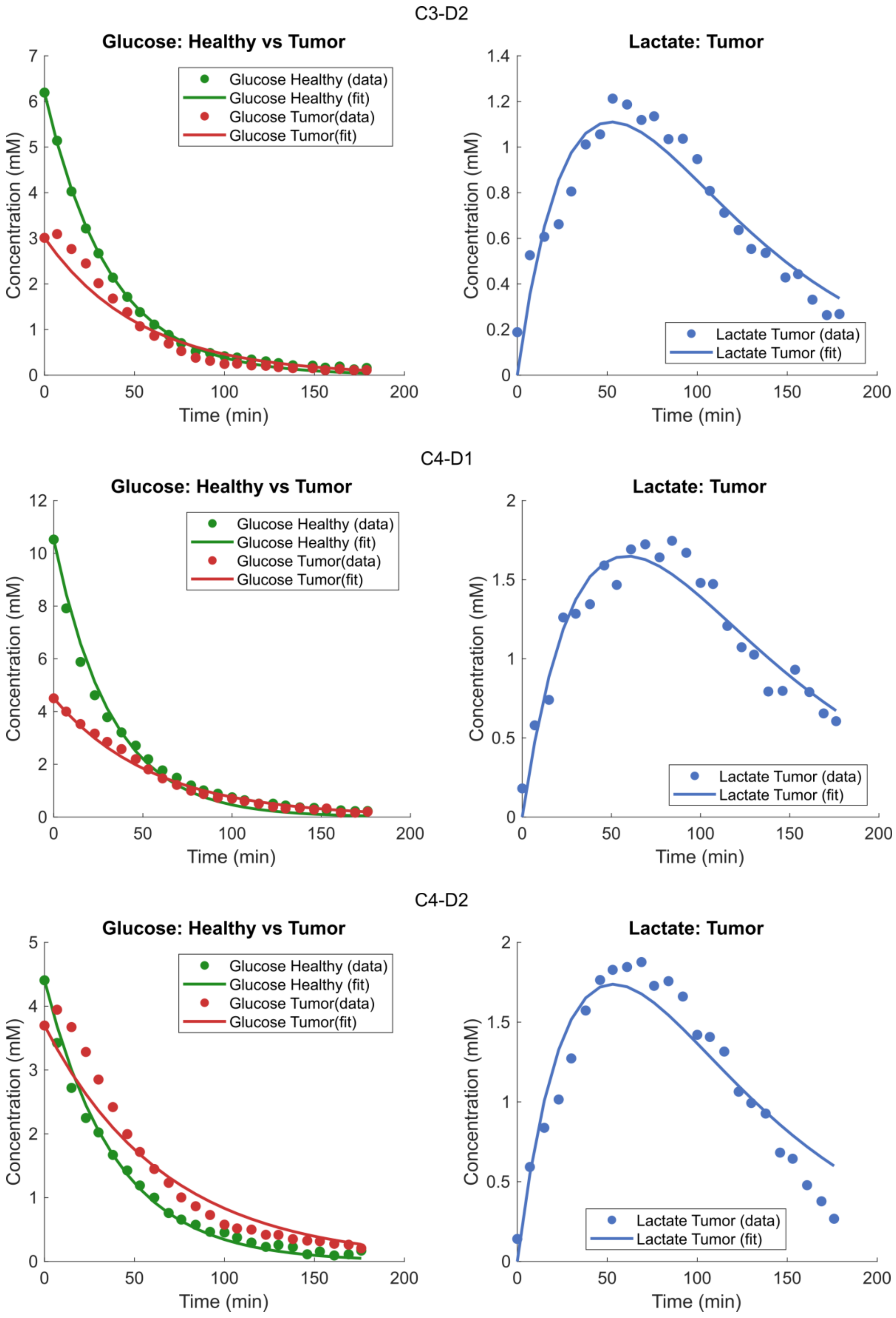
C3-D2
Glucose: Healthy vs Tumor
Glucose Healthy (data)
Glucose Healthy (fit)
Glucose Tumor(data)
Glucose Tumor(fit)
Concentration (mM)
Time (min)
Lactate: Tumor
Lactate Tumor (data)
Lactate Tumor (fit)
Concentration (mM)
Time (min)
C4-D1
Glucose: Healthy vs Tumor
Glucose Healthy (data)
Glucose Healthy (fit)
Glucose Tumor(data)
Glucose Tumor(fit)
Concentration (mM)
Time (min)
Lactate: Tumor
Lactate Tumor (data)
Lactate Tumor (fit)
Concentration (mM)
Time (min)
C4-D2
Glucose: Healthy vs Tumor
Glucose Healthy (data)
Glucose Healthy (fit)
Glucose Tumor(data)
Glucose Tumor(fit)
Concentration (mM)
Time (min)
Lactate: Tumor
Lactate Tumor (data)
Lactate Tumor (fit)
Concentration (mM)
Time (min)

C5-D1

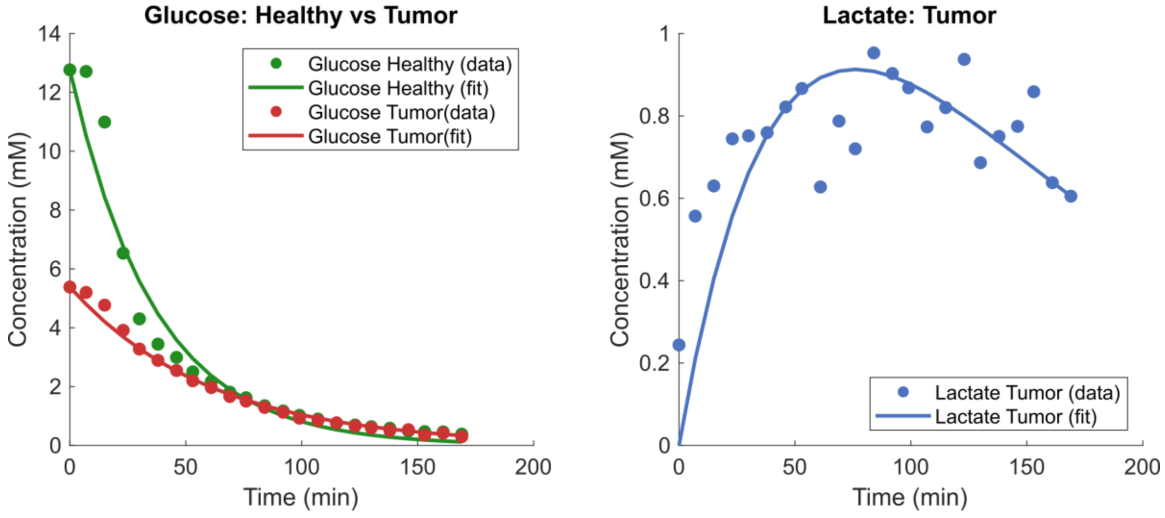

C6-D1

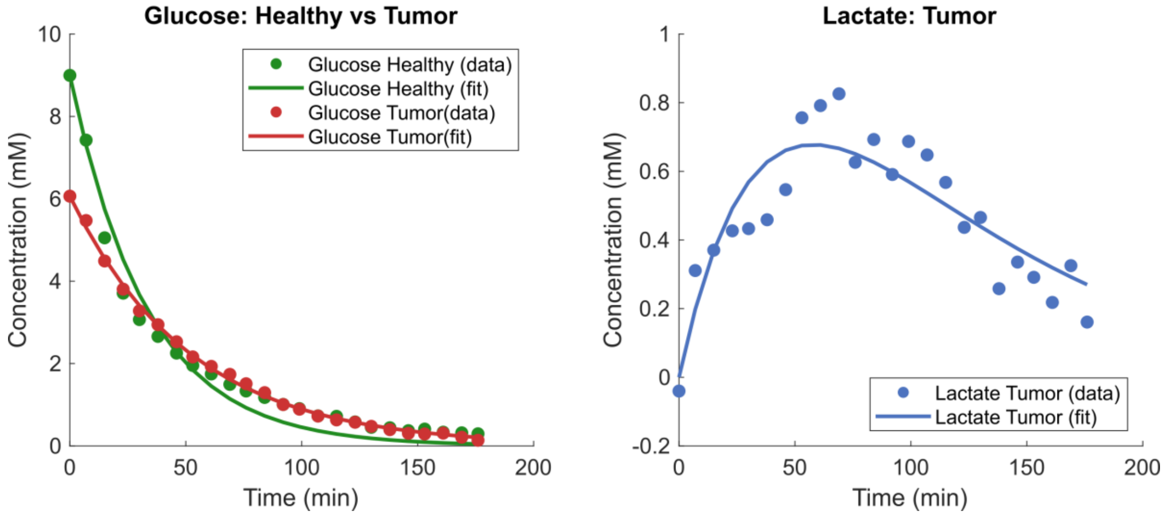

C6-D2

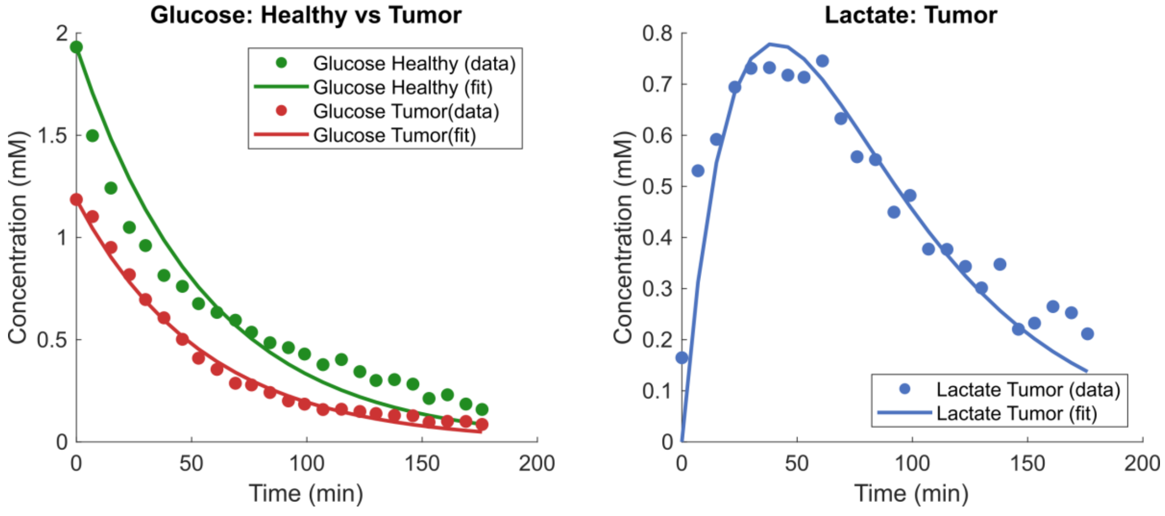

Section 4: Listing of all fitting parameters emerging from the metabolic studies.

Table S2: Estimated parameters from the fits presented in Section 2, for all the animals in both groups (treat and control) and for all successful DMI scans.

	k_{out}^{L}	k_{met}	$k_{out}^{G_t}$	$k_{out}^{G_h}$	G_0^h	G_0^t
T2-D1	0.0098	0.0058	0.0265	0.0298	14.41	4.23
T3-D1	0.0171	0.0064	0.0215	0.0234	11.39	8.48
T6-D1	0.0111	0.0082	0.0123	0.0136	11.55	6.30
T7-D1	0.0280	0.0072	0.0151	0.0241	9.03	5.96
T8-D1	0.0146	0.0094	0.0113	0.0260	7.93	3.81
T9-D1	0.0209	0.0082	0.0149	0.0312	12.24	4.65
C1-D1	0.0211	0.0184	0.0084	0.0238	12.49	4.01
C2-D1	0.0091	0.0053	0.0129	0.0148	12.98	7.95
C3-D1	0.0114	0.0073	0.0156	0.0249	13.23	5.93
C4-D1	0.0162	0.0170	0.0179	0.0313	10.52	4.50
C5-D1	0.0103	0.0061	0.0165	0.0276	12.77	5.38
C6-D1	0.0155	0.0053	0.0192	0.0299	9.00	6.06
T1-D2	0.0204	0.0121	0.0126	0.0255	2.59	3.47
T2-D2	0.0150	0.0067	0.0133	0.0199	5.49	3.68
T3-D2	0.0205	0.0124	0.0119	0.0244	5.97	3.82
T5-D2	0.0130	0.0100	0.0106	0.0171	8.30	3.92
T6-D2	0.0431	0.0241	0.0091	0.0158	5.85	3.51
T7-D2	0.0263	0.0356	0.0274	0.0431	6.11	3.31
T9-D2	0.0108	0.0078	0.0132	0.0206	9.86	4.58
C1-D2	0.0119	0.0233	0.0169	0.0243	10.66	3.26
C3-D2	0.0195	0.0192	0.0188	0.0279	6.19	3.01
C4-D2	0.0232	0.0242	0.0149	0.0255	4.41	3.70
C6-D2	0.0330	0.0450	0.0181	0.0177	1.93	1.19
T7-D3	0.0159	0.0138	0.0168	0.0306	8.83	4.55
T9-D3	0.0188	0.0219	0.0128	0.0216	7.49	2.48
